# Effect of medical school initiatives on help seeking for mental health problems among medical students: a systematic review and meta-analysis

**DOI:** 10.1136/bmjopen-2025-111351

**Published:** 2026-02-09

**Authors:** Amy E Manley, Rachel Perry, Paul Moran, Sarah Dawson, Lucy Biddle, Jelena Savović

**Affiliations:** 1 Population Health Sciences, University of Bristol, Bristol, UK; 2 NIHR Applied Research Collaboration West (ARC West) at University Hospitals Bristol and Weston NHS Foundation Trust, Bristol, UK

**Keywords:** MENTAL HEALTH, MEDICAL EDUCATION & TRAINING, Schools, Medical, Stress, Psychological, Systematic Review, PSYCHIATRY

## Abstract

**Objectives:**

Many medical students with mental health problems do not seek help. However, it is unclear what medical schools can do to promote help seeking. We sought to establish the effect of medical school initiatives on help seeking for mental health problems among medical students.

**Design:**

A systematic review and meta-analysis of studies published between 2013 and 2023.

**Data sources:**

MEDLINE Ovid, EMBASE Ovid, PsycINFO Ovid, Web of Science, ERIC, BEI and Education Abstracts.

**Eligibility criteria:**

Studies that assess the effect of an intervention delivered by a university or healthcare organisation on medical students’ attitudes towards help seeking or their help-seeking behaviour for mental health problems.

**Data extraction and synthesis:**

Two reviewers independently screened studies for inclusion and extracted data. Risk of bias was assessed using Cochrane Risk of Bias 2 (for randomised controlled trials (RCTs)) and Risk Of Bias In Non-randomized Studies of Interventions (for non-randomised studies). Studies were grouped according to intervention type. Meta-analysis was conducted using random-effects models. Certainty of evidence was assessed using Grading of Recommendations Assessment, Development and Evaluations.

**Results:**

The evidence from the meta-analyses was of very low to low certainty. Improvements in help seeking were noted in the meta-analyses of pre-post studies investigating the effect of interventions with a lived-experience component (five studies, n=492, standardised mean difference (SMD) 0.62, 95% CI 0.33 to 0.91, p<0.001); of educational interventions (two studies, n=260, SMD 0.38, 95% CI 0.01 to 0.74, p=0.04); and of interventions to improve access to services (two studies, n=1120, OR 1.80, 95% CI 1.12 to 2.88, p=0.02). Effects on help seeking diminished over time. The meta-analysis of three pre-post studies (n=677) evaluating the effect of clinical clerkships found no benefit on personal help-seeking attitudes (SMD 0.21, 95% CI −0.08 to 0.51, p=0.16). Meta-analysis of controlled studies comparing different approaches did not find superiority of face-to-face lived-experience interventions over active control interventions.

**Conclusions:**

Overall, the evidence is of very low to low certainty, due to the serious risk of bias in the included studies, most of which used uncontrolled pre-post designs. Interventions with a lived-experience component may improve medical students’ help-seeking attitudes. Standard clinical clerkships did not appear to impact personal help seeking, despite multiple previous studies suggesting they reduce stigma, suggesting barriers to help seeking extend beyond stigma and mental health literacy in this student population. Further high-quality research, particularly RCTs with long-term follow-up, is needed to firm up the evidence base in this area.

**PROSPERO registration number:**

CRD42024319771.

**STRENGTHS AND LIMITATIONS OF THIS STUDY**

- This systematic review was conducted in accordance with the Cochrane Handbook and reported in line with the Preferred Reporting Items for Systematic Reviews and Meta-Analyses 2020 guidelines. The protocol was registered on PROSPERO, ensuring methodological rigour and transparency.
- Screening, data extraction and systematic assessment of risk of bias were completed by two reviewers independently, enhancing the accuracy and reliability of the data.
- The broad search strategy across multiple databases aimed to identify all relevant evidence; however, the exclusion of non-English language studies may have led to the exclusion of some relevant findings.
- The majority of included studies were uncontrolled pre-post designs, which were at serious risk of bias, mainly due to potential for confounding.
- While three of the included studies assessed longer term change, the majority only included a single follow-up point.

## Introduction

 Studying at a medical school is demanding, involving learning a large volume of content, challenging assessments, and exposure to human suffering, sickness and death, in addition to other ‘regular’ stressors of student life, such as financial strain.^1^ These demands contribute to a high prevalence of mental health difficulties among medical students, with over a quarter of students experiencing mental health problems including anxiety and depression.^2^ Suicidal ideation among medical students is unfortunately not uncommon, affecting over one in 10 students.^2^ The nature and severity of mental health problems naturally fluctuate over the course of the medical programme, with high-stakes exams and transition points, such as the initial entry into medical school, or the shift from preclinical to clinical years, being periods of heightened stress.^3 4^ These difficulties have serious implications, potentially resulting in burnout and dropout from training.^5 6^ Furthermore, mental health problems and students’ responses to these often persist into their working lives as doctors, with implications for patient care including reduced empathy, absenteeism, increased patient safety incidents and reduced patient satisfaction.^7^,^10^

Despite concerns about the impact of mental health problems among medical students, international estimates suggest that fewer than one in six depressed medical students seek treatment.^2^ The evidence for pharmacological and non-pharmacological treatments for mental disorder in the general population is robust and relevant to medical students, yet a lack of help seeking results in undertreatment.^2 11 12^ This, in turn, leads to escalating risks and prolongation of symptoms, with duration of untreated illness correlating with worse clinical outcomes.^13^,^15^ Research within the broader student population highlights that poor mental health literacy may lead to a lack of recognition of mental illness, or awareness of how to seek help, in addition to stigmatised attitudes discouraging disclosure.^16^,^18^ Within the medical student population, specific barriers have been identified, including concerns about career implications, perceptions that doctors should be caregivers and not patients, and busy work schedules.^11 12 19 20^ These may explain why rates of help seeking among medical students are about the same as those for other student groups, despite the possession of better mental health literacy.^21^

Non-targeted approaches to promote well-being among medical students cannot replace the need for individualised treatment among students with mental health problems. Therefore, some universities have focused cohort-wide strategies on reducing stigma and promoting appropriate help seeking. Indeed, reviews of help-seeking initiatives in non-medical populations suggest that initiatives that are designed to enhance motivation can improve mental health literacy and reduce stigma, and thereby encourage help seeking among at-risk groups.^22^ However, these review findings may not be generalisable to the medical student population.^22^ Therefore, the question of how to encourage help seeking among this student group remains unanswered.

With this gap in mind, we set out to establish whether initiatives delivered to unselected medical students, in a university setting, influence the rate of help seeking for mental health problems among medical students. We sought to evaluate the direction of the effect and whether it was sustained over time.

## Methodology

This systematic review adhered to the guidance set out in the Cochrane Handbook^23^ and Preferred Reporting Items for Systematic Reviews and Meta-Analyses 2020 guidelines for reporting systematic reviews. The review protocol was registered on PROSPERO (reference CRD42024319771) and was not amended. Ethical approval was not required for this study as it is a systematic review and meta-analysis of publicly available data.

### Eligibility criteria

To be included in the review, the study had to meet the following eligibility criteria:

1. Population consisting of at least 80% medical students (students pursuing a medical degree leading to qualification as a doctor). Studies of mixed populations that provided results for subgroups of medical students which could be extracted and analysed separately were also included.
2. Intervention that may influence help seeking for mental health problems in higher education settings delivered by university or healthcare organisations. This included a breadth of initiatives, such as antistigma campaigns, and also key components of the curriculum (including clinical placements), which might directly or indirectly influence help seeking. An inclusive approach was adopted to optimise the yield of useful information for those designing and delivering medical curricula.
3. Studies using any type of comparator, including active interventions or standard practice, as well as pre-post studies without a comparator group (also referred to as uncontrolled before-after studies) were eligible.
4. There were no restrictions to eligibility based on specific study designs. Studies using any design and meeting the other inclusion criteria were considered, including randomised controlled trials (RCTs), non-randomised controlled studies, controlled or uncontrolled pre-post studies (before-after studies), interrupted time series, cohort studies and cross-sectional surveys comparing different cohorts of students.
5. The outcome of interest was help seeking for mental health problems from a professional (as opposed to from informal networks such as friends or family). Measures of attitudes towards help seeking or actual help-seeking behaviour were included. Attitudinal measures that assessed help seeking from a range of sources, including professional services, were included as evaluations of the psychometric properties of such scales (eg, the Opening Minds Stigma Scale for Health Care Providers (OMS-HC)) that have found satisfactory correlations between professional help-seeking items and the wider help-seeking factor.^24 25^ The review focused on help seeking for mental health problems prevalent among medical students (encompassing depression, anxiety disorders, obsessive-compulsive disorder, post-traumatic stress disorder, eating disorders, functional disorders, personality disorders, burnout and self-harm). Studies employing a broad definition of mental health problems, or focusing on common mental illness, were included. However, studies that exclusively focused on help seeking for psychotic disorders were excluded, given their low prevalence among medical students.
6. Publication during or after 2013 (to reflect contemporary medical curricula and practices^26^) in the English language. Reviews were excluded after screening for relevant references.

### Search strategy and information sources

The search strategies (see online supplemental information) included keywords related to mental health, medical students and help seeking or disclosure. The search was limited to articles published in English from 2013 onwards. The search was executed in MEDLINE Ovid, EMBASE Ovid, PsycINFO Ovid, Web of Science, ERIC, BEI and Education Abstracts. The reference lists of included papers and relevant reviews were examined to identify any potentially eligible studies not captured in the initial database searches.

### Selection process

Screening of titles and abstracts of identified records, and subsequently of full-text articles of potentially eligible studies, was undertaken by two independent reviewers (AEM and RP), to assess their relevance against inclusion and exclusion criteria. Any disagreements between reviewers were resolved through discussion or, if necessary, by consulting a third reviewer (JS). Rayyan^27^ was used to support the screening process.

### Data collection

A standardised data extraction form was developed in Microsoft Excel and piloted to ensure consistency and accuracy in data collection. Extraction of all data was performed by two reviewers independently (AEM and RP). Discrepancies were discussed, with reference to the original paper, and a third reviewer (JS) was consulted if necessary.

We extracted study characteristics, participants’ characteristics (including evidence of mental illness), intervention details (structured according to the Template for Intervention Description and Replication (TIDieR) Framework), the methodology employed, outcome measures, relevant findings and other details to enable risk of bias assessment. For controlled trials, change from baseline data for individual intervention arm(s) was also extracted for single-arm analysis as pre-post experiments to enable meaningful comparison with other studies. Studies were grouped according to intervention characteristics to enable exploration and comparison of initiatives with varying aims, resource requirements and modes of action.

### Risk of bias assessment

The risk of bias in included studies was rigorously assessed using appropriate tools based on their respective designs. The Cochrane Risk of Bias 2 (RoB-2) tool was employed for RCTs.^28^ The Risk Of Bias In Non-randomized Studies of Interventions version 2 (ROBINS-I v2)^29^ tool was used for non-randomised studies, and an adaptation of the ROBINS-I v2 tool described in the Cochrane Handbook^23^ was used to assess risk of bias in pre-post studies. Adapted ROBINS-I v2 assessment was also completed for assessing bias associated with pre-post data obtained from the intervention and control arms of trials. The passage of time (and therefore the possibility of exposure to cointerventions as alternative causes of observed changes) as a potential confounder was considered in the ROBINS-I assessment of pre-post studies. Two independent reviewers conducted the risk of bias assessment, with any disagreements resolved through discussion or, if needed, by a third reviewer acting as an adjudicator.

The risk of bias from missing evidence was systematically evaluated using the Risk Of Bias due to Missing Evidence (ROB-ME) tool. The assessment considered the potential for entire studies to be missing from the analysis and for the selective non-reporting of results within identified studies.

### Synthesis methods

Study details including intervention characteristics according to the TIDieR framework, direction and magnitude of change and p values were presented in tables to enable comparisons between initiatives. Pre-post methodology was the predominant design of included studies. Therefore, for controlled studies, we presented both between-group and pre-post comparison data to facilitate comparison of intervention effects across studies.

To enable comparison across different scales for continuous and ordinal outcomes (eg, attitude measures), we calculated standardised mean differences (SMD) using Hedges’ g. For the change in scores from baseline to the first follow-up point after the intervention, we used the Hedges’ g(av) method to account for the paired nature of these data.^30^ Where non-parametric tests were presented, Hedges’ g was calculated from the Wilcoxon signed-rank test p value,^31^ to avoid the sources of error that may have arisen when estimating means and SDs from median and IQR. Means and SDs for studies that reported medians and IQRs were calculated using the Wan method^32^ for presentation in table 1, but were not used in the meta-analysis. Opening Minds Scale scores were multiplied by −1 to convert to SMDs, as described in the Cochrane Handbook^23^ information on effect sizes.

**Table 1:** Effect of the interventions on health-seeking behaviours and attitudes

Study	Intervention type	Measure of help seeking	Comparator	Baseline score	Postintervention score	Score at later follow-up	P value for change over time	P value for comparison across groups
Almadani *et al*^37^	4-week psychiatry clinical clerkship	OMS-HC-15 help-seeking domain	No comparator	12.10 (3.12)	12.30 (3.22)	–	0.477*	–
Fernandez *et al*^35^	One-off lecture plus session with people with lived experience of MHP (delivered in person vs via video)	OMS-HC-15 help-seeking domain	Lecture plus F2F contact	12.58 (0.37)†	9.84 (0.40)†	11.78 (0.39) (1 month postintervention)	<0.001 for each arm‡ (significant improvement at first follow-up, but not at 1 month)	N.S.§
			Lecture plus video	11.94 (0.37)†	9.54 (0.40)†	11.65 (0.40) (1 month postintervention)		
Jarvie *et al*^39^	One-off session with comedian with lived experience of MHP	OMS-HC-20 help-seeking domain	No comparator	3.05 (0.56)¶	3.01 (0.66)¶	–	0.51*	–
Kurki *et al*^41^	Educational programme	Unvalidated 5-item help-seeking questionnaire modified from Mental Health Literacy Questionnaire by Kutcher *et al*^55^	No comparator	21.6 (2.5**)	22.1 (2.5**)	21.9 (3.8**) (2 months postintervention)	<0.001†† (from baseline to postintervention) p=0.39§ at 2 months postintervention	–
Martin *et al*^44^	One-off educational session with physicians with lived experience of MHP	OMS-HC-15 help-seeking domain	No comparator	14.4 (3.1)	12.9 (3.3)	–	<0.001*	–
Newton-Howes *et al*^36^	Psychiatry clerkship codesigned/delivered by people with lived experience of MHP (compared with standard psychiatry clerkship)	OMS-HC-15 help-seeking domain	Year 5 service user-led clerkship	11.33 (2.27)‡‡	10.33 (2.27)††	–	0.03§§	0.03¶¶
			Year 5 standard clerkship	11 (3.03)‡‡	11 (3.03)‡‡	–	0.079§§	
			Year 6 service user-led clerkship	10.67 (2.28)‡‡	10 (3.04)‡‡	–	0.0002§§	0.39¶¶
			Year 6 standard clerkship	10.33 (2.29)‡‡	10.33 (2.29)‡‡	–	0.075§§	
Seritan *et al*^38^	Opening a point of contact for students to access support and offering educational sessions	Uptake of university-based psychological support services	Proportion of students using service	32.11546 (6.944)	40.175 (4.392)	–	0.004***	–
			Rate of change in service use	1.842727 (1.468)	1.55 (1.468)	–	0.828***	
Smith^43^	Campaign plus sessions with senior students with lived experience of MHP	Formal resources domain−General Help-Seeking Questionnaire	No comparator	2.63 (NR)†††	3.00 (NR)†††	–	N.S.*	–
Takahashi *et al*^42^	One-off educational session	Formal resources domain-General Help-Seeking Questionnaire	No comparator	10.73 (4.62)	13.43 (4.64)	11.67 (4.70) (6 months postintervention)	<0.001‡ N.S. 6 months postintervention	–
Williams and Davis^45^	Email offering appointments to students	Uptake of student mental health services (%)	No comparator	8.3%	17.0%	–	<0.001‡‡‡	–
Zavorotnyy *et al*^40^	Psychiatry clinical clerkship	OMS-HC-20 disclosure subscale	No comparator	13.9 (3.7)	13.3 (3.5)	–	<0.001	–

* Paired t-test (as originally reported in the paper).

† Unusually small SDs reported in the paper; therefore, SD=2.9 (obtained from a large study, n=1523^24^) was used to calculate SMD before adjustment for sample size as per the Hedges’ g (adjusted) method.

‡ One-way repeated measures ANOVA (as originally reported).

§ Mixed model MANOVA (as originally reported).

¶ Scores represent mean (SD) score on help-seeking items (as originally reported) rather than subscale total.

** SD calculated from SE and n.

†† Repeated measures ANOVA adjusted for year, gender, moved from elsewhere and year when they started their studies (as originally reported).

‡‡ Estimated from median (IQR) via Wan method.^32^

§§ Wilcoxon signed-rank test.

¶¶ Wilcoxon rank-sum test.

*** Calculated using segmented logistic regression based on information provided in the paper for all available years and accounting for multiple service use of 7% postintervention.

††† Means calculated from item-level results. SDs could not be accurately calculated for each group; therefore, the SD (0.52) provided for the whole sample was used to calculate SMD.

‡‡‡ Calculated using χ2 test based on information provided in the paper.

ANOVA, analysis of variance; F2F, face to face; MANOVA, multivariate analysis of variance; MHP, mental health problem; NR, not reported; N.S., non-significant at the p<0.05 level; OMS-HC, Opening Minds Stigma Scale for Health Care Providers; SMD, standardised mean difference.

For dichotomous outcomes, such as uptake of services (ie, expressed as a proportion of all students using services), we calculated ORs and 95% CIs. Where interrupted time series data were available, ORs were calculated accounting for pre-existing trends in service uptake (ie, using all available datapoints) using segmented logistic regression.^33^ This enabled calculation of ORs for both the immediate change in uptake of services on introduction of the intervention (level change) and change in the rate of change in uptake following the intervention (slope change).^33^

Random-effects meta-analyses (restricted maximum likelihood method) were conducted to synthesise findings from studies of comparable initiatives and presented visually using forest plots. Sensitivity analysis using fixed-effects models was conducted for meta-analyses of identical interventions with low heterogeneity. Analyses were undertaken in the software package Stata V.18.^34^

Sensitivity analyses by risk of bias, according to the ROBINS-I tool, were conducted. However, funnel plots to evaluate the risk of publication bias could not be performed due to the small number of studies available for each meta-analysis.^23^

### Investigation of heterogeneity

Intervention components, study designs and population characteristics (illness prevalence and gender) were tabulated to evaluate the effect of these characteristics on intervention effects. To aid comparison of studies by illness severity, the documented prevalences of mental illness are presented. Where an ordinal measure of symptomatology was provided (eg, the Patient Health Questionnaire 9), standardised cut-off scores were used to estimate prevalence of mental health problems within the population. Meta-regression was not possible due to fewer than 10 studies reporting these variables, rendering such analysis unreliable.^23^

### Certainty of evidence

Assessment of the certainty of the evidence was based on the Grading of Recommendations Assessment, Development and Evaluations (GRADE) approach, as outlined in the Cochrane Handbook chapter 14.^23^ This considers risk of bias as evaluated by RoB-2 or ROBINS-I, inconsistency in the results which cannot be explained by modifiers (eg, intervention type), indirectness in the measurement of the outcome in medical students, imprecision and publication bias. Imprecision of the estimate of effect was graded based on the number of included studies, participants and the width of the CIs. Two thresholds, reflecting the minimal important difference (defined as ≤−0.2 or ≥+0.2 SDs for continuous outcomes, and the equivalent ORs of ≤0.7 or ≥1.4 for dichotomous outcomes), were used to assess precision of the CIs.

### Patient and public involvement

Patients and/or the public were not involved in the design, or conduct, or reporting, or dissemination plans of this research.

## Results

**Figure 1:**
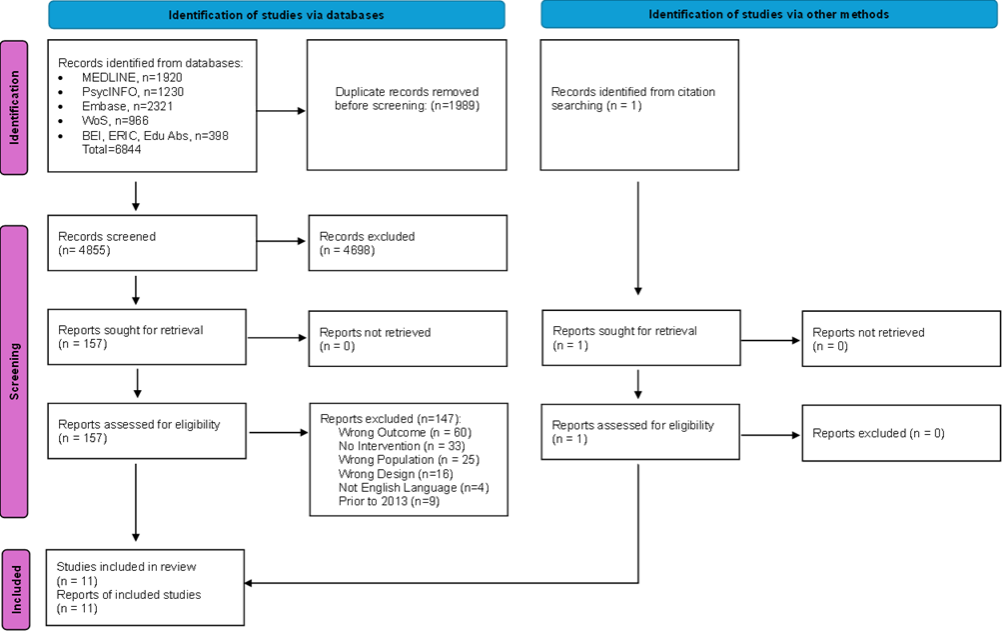
PRISMA flow diagram of included trials. BEI, British Education Index; Edu Abs, Education Abstracts; ERIC, Education Resources Information Center; PRISMA, Preferred Reporting Items for Systematic Reviews and Meta-Analyses; WoS, World of Science.

11 studies met the predefined inclusion criteria and were eligible for inclusion in the review (see figure 1). These studies included one RCT,^35^ one non-RCT,^36^ one cross-sectional survey,^37^ one interrupted time series^38^ and seven uncontrolled pre-post studies, six of which followed up a single cohort of participants over time,^39^,^44^ while one measured service uptake among a changing cohort of all enrolled medical students.^45^ Studies were conducted in Asia (4), North America (4), Europe (2) and Oceania (1). All included studies were published in English language between 2013 and 2023.

### Population characteristics

We included nine studies that assessed attitudes to help seeking (1684 participants)^35^,^3739^ and two studies reporting change in uptake of services (1120 participants).^38 45^

Six studies recruited participants in their first or second year of medical school,^3539 41^,^44^ and a further three included all students regardless of academic year.^37 38 45^ The majority of participants, for which age data were available, were in their 20s. Among the eight studies for which gender information was available, the percentage of males ranged from 22% to 74%, with a mean of 53%.^3537^,^42 44^ Ethnicity was reported by five studies using varied classifications (see table 2).^35 38 39 43^

**Table 2:** Study design and characteristics of included studies

Study: first author, country	Quantitative study design	Sample size	Eligible for participation or service use	Outcome measure of help seeking	Characteristics of populations			
					Gender (% male)	Age/academic year of study	Mental health status	Ethnicity
Almadani,^37^ Saudi Arabia	Cross-sectional survey comparing year 1–4 pre-psychiatry placement students with year 4–5 postplacement students	367	384	OMS-HC-15 help-seeking domain	51	Age NR Years 1–5	MH problem 13.6%*	NR
Fernandez,^35^ Malaysia	RCT (two active arms)	F2F=51 Video=51	111	OMS-HC-15 help-seeking domain	22	Mean 21.1 years (0.6)† Year 2	NR	81% Malay, 11% Chinese, 8% Indian
Jarvie,^39^ Canada	Uncontrolled pre-post study evaluating attitudes	49	130	OMS-HC-20 help-seeking domain	45	94% aged 20–30 years Year 2	MH problem 31%*	European 65%, Asian 18%, SE Asian 6%, Black 0%, Hispanic 0%, Other 10%
Kurki,^41^ Finland	Uncontrolled pre-post study evaluating attitudes	158	220	Unvalidated 5 item help-seeking questionnaire	74	68% aged 18–21 years Year 1	MH problem 31%† Psychiatric disorder 47.9%‡§	NR
Martin,^44^ Israel	Uncontrolled pre-post study evaluating attitudes	53	61	OMS-HC-15 help-seeking domain	54	66% aged 25–29 years Year 2	MH problem 18%† Depression 88.2%¶§	NR
Newton-Howes, ^36^ New Zealand	Non-randomised controlled trial evaluating attitudes	Codesigned clerkship=125 Standard clerkship=111	442†	OMS-HC-15 help-seeking domain	NR	Age NR Years 5 and 6	NR	NR
Seritan,^38^ USA	Interrupted time series (15 time points) evaluating health service uptake	N/A	420†	Uptake of university-based psychological support services	46	Age NR All years	NR	44% Caucasian
Smith,^43^ USA	Uncontrolled pre-post study evaluating attitudes and self-reported behaviour	15	160	General Help-Seeking Questionnaire−formal resources domain	NR**	71% aged 20–25 years Years 1–2	NR	NR**
Takahashi,^42^ Japan	Uncontrolled pre-post study evaluating attitudes	102	136	General Help-Seeking Questionnaire−formal resources domain	65	Mean 20.0 years (1.4) Year 2	Depression 1.1%¶§	NR
Williams,^45^ USA	Uncontrolled pre-post study evaluating health service uptake	N/A	Approximately 700††	Uptake of student mental health services	NR	Age NR All years	NR	NR
Zavorotnyy,^40^ Germany	Uncontrolled pre-post study evaluating attitudes	182	256	OMS-HC disclosure subscale	65	Mean 24.4 years (2.7) Years 3–4	MH problem 6%*	NR

Some studies used mixed methodology. Only the quantitative components of the studies are described and reported here.

* Self-reported mental health problem.

† Calculated from information provided in the paper.

‡ Moderate or greater symptoms as measured by the General Health Questionnaire (GHQ) >11.5.^56^

§ Calculated from mean and SD of scale scores.

¶ Moderate or greater symptoms as measured by PHQ-9>10.

** Gender and ethnicity of the 15 students included in the analysis relevant to this review were not reported.

†† Estimated from institutional website data.^57^

.MH, mental health; NR, not reported; OMS-HC-15, Opening Minds Stigma Scale for Health Care Providers 15-item version; PHQ-9, Patient Health Questionnaire-9; RCT, randomised controlled trial.

Six studies presented a baseline measure of mental health morbidity for their population, with a variety of measures being used.^3739^,^42 44^ Where this information was available, between 6% and 31% (mean 17.5%) of participants reported having had a mental health problem, and between 1.1% and 88.2% (mean 37.8%) scored as having moderate or greater symptoms on a validated screening tool for mental health problems.

### Intervention characteristics

The interventions are described in accordance with the TIDieR framework. Each intervention is summarised in online supplemental table 4. Interventions employed diverse strategies and have been presented in four broad categories: clinical clerkships; initiatives with a lived-experience component; service delivery and outreach; and educational initiatives aimed at improving mental health literacy and enhancing coping skills. Initiatives with a lived-experience component refer to those in which people who had personal experience of mental health problems delivered part of the intervention, for example, by sharing their personal experience of mental health problems with students or codesigning and delivering workshops on recovery. Although clinical clerkships are primarily educational and are not specifically designed to promote help seeking, studies have investigated whether such clerkships are associated with attitudinal change,^36 37 40^ and thus they were included.

The initiatives varied in duration and intensity, ranging from an email sent once per semester,^45^ to a one-off educational session delivered over 3 hours,^35^ to recurrent interventions delivered over a 5-week clinical clerkship.^36^ Nine interventions involved a component that was delivered face to face. Seven of the interventions were either compulsory or were associated with course credit.

### Impact on help seeking

Nine studies assessed help-seeking attitudes, one of which additionally assessed behaviour, as measured through self-reported questionnaires. Two studies evaluated utilisation of mental health services. Seven studies reported positive effects of interventions on help-seeking behaviour or attitudes over time (table 1).

### Comparison between groups

**Figure 2:**
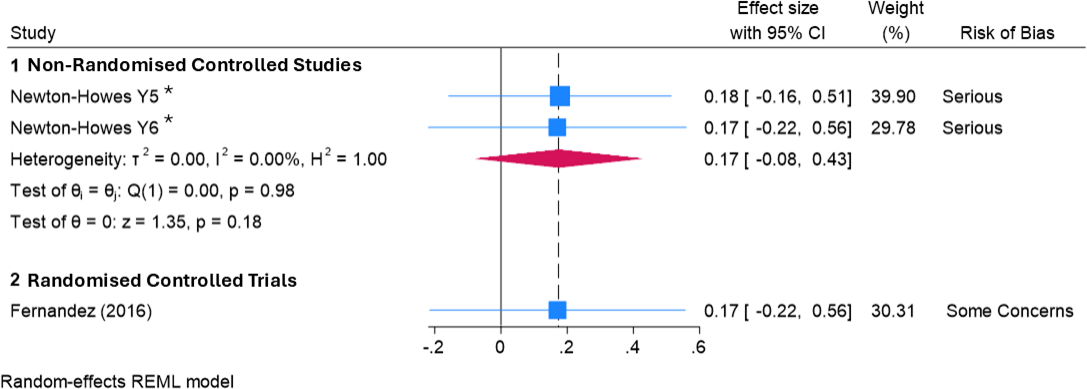
Forest plot demonstrating the effect of face-to-face interventions delivered by people with lived experience of mental illness on medical students’ attitudes to help seeking for personal mental health problems when compared with control interventions. *Study presents data from two non-randomised controlled studies each with an intervention and a control group. These groups contained participants from different years of study, and participants could not be in more than one group. REML, restricted maximum likelihood.

Two studies with a comparative design^35 36^ assessed the effect of lived-experience initiatives. The first study^35^ was a one-off intervention that included a person who had experienced severe mental illness, sharing their experience of recovery with a group of 51 students. The second study^36^ involved a codesigned clinical clerkship that included a service user-led workshop about stigma and recovery. The control groups in both studies received active comparator interventions, which also had the potential to influence help-seeking attitudes (ie, a pre-recorded video of a personal testimony about the experience of illness^35^ and a standard clinical clerkship,^36^ respectively). Face-to-face personal testimony of illness was not found to be statistically superior to the video-based control. There was no evidence that the effect of the coproduced clinical clerkship differed from the standard clerkship. This low-certainty evidence suggests that these face-to-face lived-experience initiatives may result in little to no difference in help-seeking behaviour compared with video-based lived-experience initiatives (SMD 0.17, 95% CI −0.22 to 0.56, p=0.39; low-certainty evidence) or standard clinical placements (SMD 0.17, 95% CI −0.08 to 0.43, p=0.18; low-certainty evidence; see figure 2). This finding was not altered by use of a fixed-effects model for meta-analysis (see online supplemental figure 5).

### Comparison between before and after an intervention

**Figure 3:**
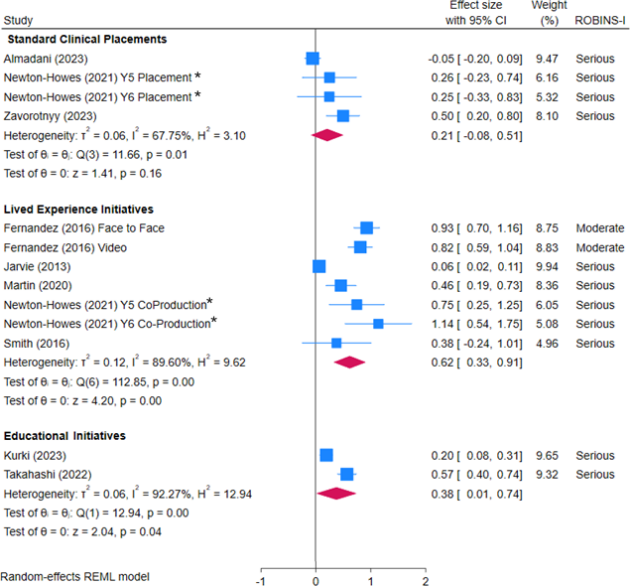
Forest plot of the effect of standard clinical placements, lived-experience interventions and educational interventions on medical students’ attitudes to help seeking for personal mental health problems, based on data from before-after study. REML, restricted maximum likelihood; ROBINS-I, Risk Of Bias In Non-randomized Studies of Interventions.

Standard clinical clerkships in psychiatry (total sample size, n=677) showed little to no benefit in changing attitudes to help seeking for mental health problems, but the evidence is very uncertain (summary estimate from three studies, SMD 0.21, 95% CI −0.08 to 0.51, p=0.16; very low-certainty evidence; see figure 3). There was substantial heterogeneity between the three studies included in this meta-analysis (I^2^=67.75%).

Pre-post data from five studies^35 36 39 43 44^ indicated that initiatives that included a lived-experience/coproduction element (total sample size, n=492) were associated with positive changes in help seeking; however, the effects were highly variable, with SMDs ranging from 0.06 to 1.14. There was low-certainty evidence that initiatives with a lived-experience component may improve help-seeking attitudes compared with baseline, based on a summary estimate from five studies (SMD 0.62, 95% CI 0.33 to 0.91, p<0.001; see figure 3), with substantial heterogeneity (I^2^=89.60%).

Very low-certainty evidence suggested that educational initiatives without a lived-experience element (n=260) also may improve help-seeking attitudes slightly (SMD 0.380, 95% CI 0.01 to 0.74, p=0.04; see figure 3), but the evidence is very uncertain. There was considerable heterogeneity between studies (I^2^=92.27%).

**Figure 4:**
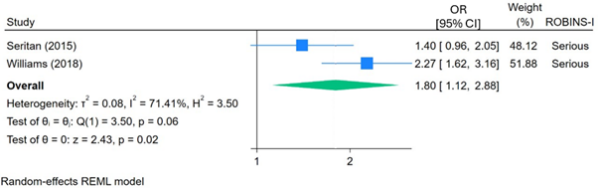
The effect of interventions to improve accessibility of services on medical students’ uptake of mental health services. REML, restricted maximum likelihood; ROBINS-I, Risk Of Bias In Non-randomized Studies of Interventions.

Findings from studies^38 45^ that involved increasing the availability or accessibility of mental health services on campus indicated that these initiatives may increase service use (OR 1.80, 95% CI 1.12 to 2.88, p=0.015; see figure 4) in the year following the intervention, but the evidence is very uncertain. Furthermore, the non-randomised nature of these studies meant it was not possible to determine whether the changes in service use were attributable to increased help seeking, or alternatively, whether they reflected changes in *where* students sought help (potentially moving from external healthcare services to university services). Seritan *et al*’s study^38^ showed that the rate of service uptake was indeed increasing, although modestly, prior to the intervention (1.83% increase per year). At the time of the intervention, there was an immediate increase of 8.44% when controlling for the pre-existing trend. However, the rate of change in service uptake then slowed slightly following the intervention (to 1.55% increase per year, p=0.828). Williams and Davis’^45^ study did not provide details of trends in service use prior to the intervention; therefore, this could not be controlled for in the meta-analysis.

### Sustained change over time

Three of the studies which provided pre-post data assessed students at more than one time point after the intervention.^35 41 42^ All showed evidence of significant improvement at the initial follow-up after the intervention. However, this improvement was not significantly sustained at 1, 2 and 6 months following the intervention.

### Subgroup and sensitivity analyses

Due to the limited reporting of demographic characteristics, meta-regression by gender or proportion of students with a mental illness was not feasible. However, these characteristics were tabulated against baseline help-seeking attitudes and SMD to enable comparison of effect size by gender and symptomatology across studies (see online supplemental tables 6 and 7).

All but two^35^ studies were deemed at serious risk of bias; therefore, sensitivity analysis excluding studies at serious risk of bias was only possible for lived-experience initiatives. This did not substantially alter the findings, revealing a similar effect size for studies at moderate risk of bias (SMD 0.59, 95% CI 0.05 to 1.13, p=0.03; see online supplemental figure 6) as the overall analysis (see figure 3).

### Risk of bias

Methodological limitations of studies including pre-post study designs, lack of randomisation, confounding, small sample sizes and high attrition rates contributed to the serious risk of bias identified in all but two studies. Risk of bias assessments using RoB-2 and ROBINS-I v2 contributed to the GRADE assessment of certainty of evidence for each meta-analysis.

The single RCT^35^ had a lower risk of bias compared with non-randomised studies and pre-post studies, with ‘some concerns’ being raised due to a lack of a published protocol with analysis plan.

The ROBINS-I assessment revealed serious concerns for all except two studies.^35^ Details of the rationale for risk of bias assessments are displayed in online supplemental table 5. Given the time that elapsed between measures during pre-post studies, confounders that were not measured or controlled for may have arisen during this period. Differences in population or comparator groups may have given rise to confounding in comparative studies. Six studies^3640^,^44^ were deemed to be at serious risk of bias due to missing data, with fewer than 90% of participants being followed up. Two studies^37 43^ described multiple measures of help seeking, though there was little evidence this resulted in selective reporting of results. Conversely, Williams and Davis^45^ presented multiple analyses of data pertaining to service uptake, suggestive of serious risk of bias arising from selective reporting of findings. No concerns were raised about the classification of the intervention or participant selection. Only Smith*’*s^43^ study, which excluded participants who did not adhere to the intervention (ie, per protocol analysis), raised concerns about bias due to deviations from the intended intervention.

Assessment of publication bias using the ROB-ME framework^46^ suggested low risk of publication bias for meta-analyses of clerkship, lived-experience or educational initiatives. While the included studies lacked prepublished protocols, there was no indication that results were selectively withheld from the reports. Help seeking was not the primary outcome in the majority of included studies in the first three meta-analyses; therefore, publication decisions were less likely to be driven by this outcome of interest. Furthermore, none of the meta-analyses were dominated by small, highly significant studies. Therefore, it was deemed less likely that entire studies were missing from these meta-analyses due to the direction or statistical significance of their findings. For the meta-analysis of initiatives to improve access to services, the primary outcome of included studies was help seeking, and there was possible selective presentation of findings in one study.^45^ Therefore, the possibility of non-publication of relevant findings based on significance or direction was higher and the likelihood of the risk of publication bias for this study was deemed to raise some concerns. Nonetheless, given the limited number of studies and their observational nature, without registered protocols, there may be further similar unpublished studies.

### Certainty of the evidence

Certainty of evidence gradings is shown in table 3. Certainty of evidence for most meta-analyses was downgraded due to serious risk of bias. The meta-analyses of controlled studies of initiatives with a lived-experience component were also downgraded due to an imprecise estimate of effect, leading to a grading of low-certainty evidence. The meta-analyses in which the majority of studies adopted a pre-post design were all downgraded due to substantial inconsistency given the heterogeneity of studies. This led to a grading of low-certainty evidence for the meta-analysis of lived-experience initiatives which included pre-post data. Additionally, the meta-analyses of educational, standard clinical placement and access to services initiatives, which each combined fewer than five studies, were downgraded due to imprecise estimates, leading to a grading of very low certainty for each.

**Table 3:** GRADE assessment of evidence summary of findings

Certainty assessment							Patients (n)		Effect	Certainty
Studies (n)	Study design	Risk of bias	Inconsistency	Indirectness	Imprecision	Other considerations	Intervention or follow-up	No intervention (control) or baseline	Effect size (95% CI)	
Effect of interventions with a face-to-face lived-experience component on attitudes to help seeking (comparison data)										
1	RCT	Not serious	Not serious	Not serious	Very serious*	None	51	51	SMD **0.17 SD higher††** (0.22 lower to 0.56 higher)	⨁⨁◯◯ Low*
2	Non-randomised controlled trial	Serious†	Not serious	Not serious	Serious‡	None	125	111	SMD **0.17 SD higher††** (0.08 lower to 0.43 higher)	⨁⨁◯◯ Low†‡
Effect of psychiatric placements on attitudes to help seeking (pre-post data)										
4	Non-randomised studies	Serious†	Serious§	Not serious	Serious‡	None	477	476	SMD **0.21 SD higher††** (0.08 lower to 0.51 higher)	⨁◯◯◯ Very low†‡§
Effect of interventions with a lived-experience component on attitudes to help seeking (pre-post data)										
6	Non-randomised studies	Serious†	Serious§	Not serious	Not serious	None	295	295	SMD **0.62 SD higher††** (0.33 higher to 0.91 higher)	⨁⨁◯◯ Low†§
Effect of educational interventions on attitudes to help seeking (pre-post data)										
2	Non-randomised studies	Serious†	Very serious§	Not serious	Serious‡	None	260	260	SMD **0.38 SD higher**†† (0.01 higher to 0.74 higher)	⨁◯◯◯ Very low†‡§
Effects of improving access to services on service uptake (pre-post data)										
2	Non-randomised studies	Serious†	Serious¶	Not serious	Serious‡	Reporting bias**	273/1120 (24.4%)	186/1120 (16.6%)	OR **1.80** (1.12 to 2.88)	⨁◯◯◯ Very low†‡¶**

* Downgraded as 95% CIs for the overall effect are wide and encompass negative effect sizes (<−0.2) as well as negligible and positive effect sizes (>0.2), demonstrating very serious imprecision of estimates.

† Downgraded as the majority of studies were at serious risk of bias, as assessed using ROBINS-I.

‡ Downgraded as 95% CIs for the overall effect are wide and encompass negligible (SMD −0.2 to 0.2 or OR ≤0.7 to ≥1.4) as well as positive (SMD>0.2 or OR>1.4) effect sizes, demonstrating imprecision of estimates.

§ Downgraded due to considerable (I2>60%) to substantial (I2>90%) heterogeneity. However similar populations, comparators and outcome measures within categories suggest heterogeneity is likely to arise from variation in intervention characteristics. Direction of study effects are also similar within catagories.

¶ Downgraded due to considerable (I2>60%) heterogeneity, with heterogeneity in intervention characteristics likely being compounded by study design (interrupted time series vs before/after design), with Williams’ data not accounting for pre-existing trends in service uptake.

** Assessed using the ROB-ME framework.

†† Difference in SMD (as measured in standard deviations) as compared to the comparitor group (for controlled studies) or baseline scores (for pre-post studies)

GRADE, Grading of Recommendations Assessment, Development and Evaluations; RCT, randomised controlled trial; ROBINS-I, Risk Of Bias In Non-randomized Studies of Interventions; SMD, standardised mean difference.

## Discussion

This systematic review synthesised evidence on the effect of medical school initiatives on help seeking for mental health problems among medical students. The meta-analysis indicated that initiatives incorporating a lived-experience component may improve help-seeking attitudes (low-certainty evidence). Lesser effects were seen with educational initiatives without a lived-experience element and initiatives to promote accessibility (very low-certainty evidence). However, CIs ranged from negligible to moderate effects, and the evidence is very uncertain. Controlled trials compared active interventions. The evidence suggests that face-to-face lived-experience intervention results in little to no difference in help-seeking attitudes compared with control interventions. In studies with multiple follow-up time points, benefits were not sustained over time.^36 37 40^

### Strengths and limitations of the evidence base

Most of the included studies were at serious risk of bias and the certainty of evidence was low to very low across the intervention types, primarily due to this risk of bias. The majority were uncontrolled pre-post designs, which are heavily susceptible to confounding and social desirability bias, potentially artificially inflating effect estimates. It is notable that those studies that included active comparators found no significant difference between the interventions, and we cannot determine to what degree any detected change is due to time, unreported co-occurring events or social desirability. Furthermore, missing data due to attrition or non-reporting of response rates in six studies raised concerns that participants who did not complete the study may have differed systematically from those who did. Therefore, reported changes to help seeking cannot be definitively attributed to the intervention itself.

The small sample sizes and small number of studies included in the meta-analyses resulted in wide CIs, leading to uncertainty about the direction of some effects and limiting conclusions about the size of any effect. Furthermore, attitudinal change may not translate into behavioural change. The validated attitudinal measures used by some studies (eg, OMS-HC) have been shown to be associated with help-seeking behaviour.^47^ However, the intention-behaviour gap is well recognised^48^; therefore, the effect of these initiatives on behaviour remains uncertain. Most studies in this field did not register their prepublication protocol, and the degree of selective non-publication of relevant studies is difficult to quantify. However, as help seeking was a secondary measure in most studies, non-significant findings in this domain were less likely to influence likelihood of publication, reducing the likelihood of publication bias. Finally, the results from three studies that assessed longer term impact on attitudes indicated that change is not sustained over time. These limitations constrain the practical relevance of the findings and highlight the need for rigorous evaluations to identify which initiatives are most effective and what frequency can foster lasting impact.

### Strengths and limitations of the review process

Our meta-analysis was conducted in accordance with methodology outlined in the Cochrane Handbook.^23^ We developed a broad search strategy and searched multiple databases to reduce the likelihood of publication bias, which was evaluated using the validated ROB-ME framework. We excluded non-English language studies that risk excluding relevant findings. However, the four non-English language articles reviewed during the full-text screening were ineligible for other reasons. Double screening of abstracts and full texts and data extraction completion by two researchers ensured data accuracy and reliability. However, by grouping studies according to intervention type, we were able to conduct focused meta-analyses of similar initiatives, enabling comparison between intervention types. We did this in order to optimise the utility of our findings to educators and academics. We prospectively opted to use random-effects meta-analysis, given variation in help-seeking initiatives, which would make a single common treatment effect unlikely.^49^ Nonetheless, we recognise such models have been criticised for the difficulty in accurately estimating the between-study variance when the number of included studies is small, which can affect the precision of the CIs.^49^

### Implications for practice

Initiatives designed to encourage access (eg, informative emails offering appointments) may be effective in medical students, as has been suggested in other populations.^50^ However, the meta-analytical evidence from the studies presented here is very uncertain. Such initiatives may reduce the effort required to seek help^45^ and address common concerns among students^20^ by tacitly suggesting that needing and seeking help is common and appropriate in the medical student population. Our very low-certainty finding that standard psychiatry clerkships have little to no effect on help-seeking attitudes contrasts with empirical and meta-analytical evidence, including from studies within this review, that clinical placements reduce medical students’ stigma towards people with mental health problems.^35 36 40 51 52^ This suggests that, contrary to findings in other populations,^53^ addressing stigma in medical students may be insufficient to change help-seeking behaviour in this population. This may reflect some unique barriers faced by medical students, including their concerns about career implications of seeking help for mental health problems.^11 12^ It could also reflect medical students’ deeply engrained attitudes about needing to retain the identity of being a ‘healer’, rather than a help seeker,^20^ which may lead to students’ considering seeking help to be less appropriate for themselves than for others.

Initiatives with a lived-experience component may improve help-seeking attitudes in the medical student population. This finding resonates with literature showing that contact with people with mental health problems reduces stigma among healthcare professionals, thus promoting help seeking.^51 54^ Furthermore, unlike standard clinical contact, codesigned lived-experience initiatives and stories of recovery may reduce the imbalance of power between patients and doctors and demonstrate that recovery is possible, potentially highlighting the value of seeking treatment for mental illness. Furthermore, initiatives that involve medical students or doctors may reassure students that they can have fruitful careers despite mental health problems.^54^

These findings tentatively suggest lived-experience initiatives may be valuable and could be integrated with mental health teaching, as well as being standalone well-being sessions within medical curricula. However, all of our findings are based on low to very low-certainty evidence, and the lack of evidence for sustained change suggests interventions will likely need to be sustained or repeated throughout medical programmes.

### Future research

Future research should prioritise more rigorous study designs, particularly RCTs with longer term follow-up, to establish causality and the sustainability of effects. The low-certainty evidence that lived-experience initiatives may improve help seeking warrants further research to identify whether the profession of the individual with a history of mental illness (as a doctor or not) influences the effect of the intervention on help-seeking behaviour. Prospective studies that measure observed help-seeking behaviours rather than relying solely on attitudinal scales would be particularly valuable. Developing and evaluating initiatives that specifically target the unique barriers faced by medical students, such as fear of career repercussions, may be a valuable avenue of further investigation.

## Conclusion

This review synthesised the evidence about the impact of non-targeted initiatives on help seeking for mental disorders among medical students, a group for whom mental health problems are common and help-seeking rates are low. The current evidence base is of low to very low certainty, with a preponderance of pre-post designs and most studies showing serious risk of bias. The meta-analysis provides low-certainty evidence that initiatives with a lived-experience component may improve help-seeking attitudes. Educational initiatives and initiatives aiming to improve access to services may improve attitudes, but the evidence is very uncertain. Any potential benefits to help seeking appear not to be sustained over time. Further development, implementation and rigorous evaluation of approaches to promote help seeking will be valuable in supporting more medical students with mental health problems to seek help.

## Supplementary material

10.1136/bmjopen-2025-111351 online supplemental file 1

10.1136/bmjopen-2025-111351 online supplemental file 2

10.1136/bmjopen-2025-111351 online supplemental file 3

## Data Availability

All data relevant to the study are included in the article or uploaded as supplementary information.
