# Supplementary figures and images for: Effect of medical school initiatives on help seeking for mental health problems among medical students: a systematic review and meta-analysis
Source: BMJ Open. 2026 Feb 9;16(2):e111351. doi: 10.1136/bmjopen-2025-111351 (PMC12887498; doi:10.1136/bmjopen-2025-111351)

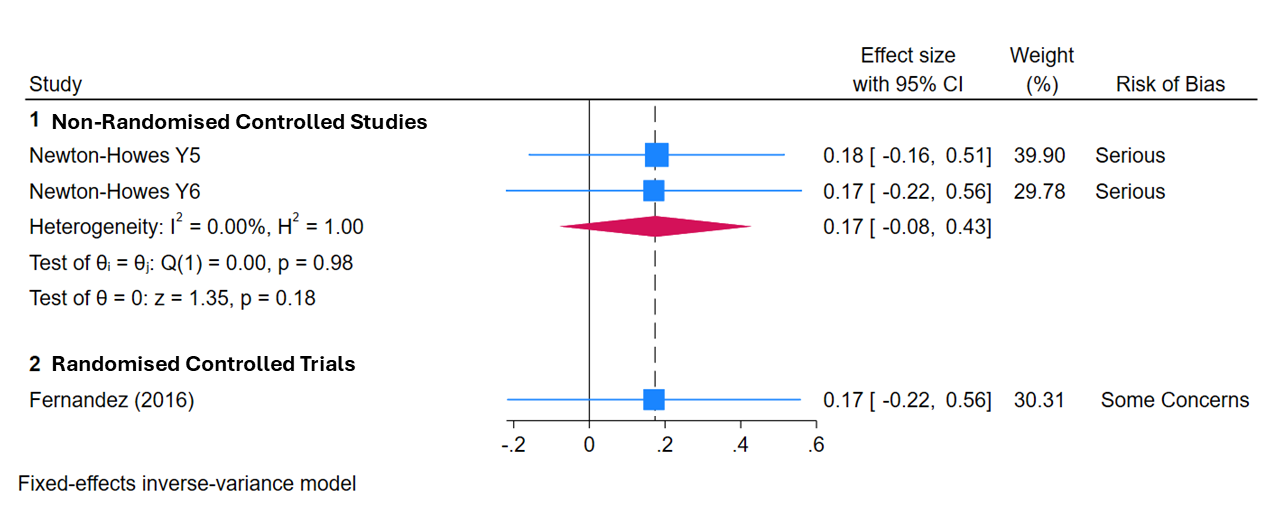

Supplement: online supplemental file 1 [file bmjopen-16-2-s001.png]

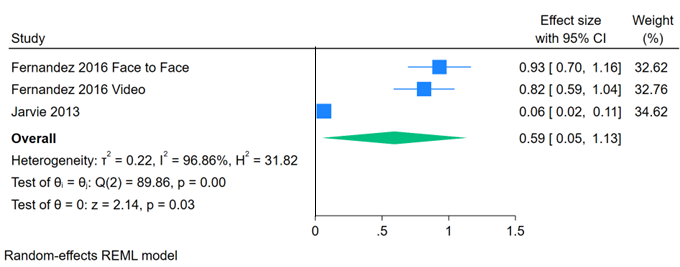

Supplement: online supplemental file 2 [file bmjopen-16-2-s002.png]
