# Supplementary material for: Effect of medical school initiatives on help seeking for mental health problems among medical students: a systematic review and meta-analysis
Source: BMJ Open. 2026 Feb 9;16(2):e111351. doi: 10.1136/bmjopen-2025-111351 (PMC12887498; doi:10.1136/bmjopen-2025-111351)
Supplement: online supplemental file 3 [file bmjopen-16-2-s003.docx]

**Supplementary Information:**

**Appendix 1: Search Strategies**

**Conducted: 16-Jan-2024**

Total, n=6810

Duplicates removed =1955

Total to screen, n= 4855

*************************************************************************

**Ovid MEDLINE^(R)^ ALL** <1946 to January 16, 2024>

1 Mental Health/ 64850

2 exp Mental Disorders/ 1459297

3 *Anxiety/ 49220

4 exp Self-Injurious Behavior/ 85147

5 Psychological Distress/ 4393

6 Stress, Psychological/ 134536

7 (mental* or psychiatr* or psychopathol*).tw,kf. 762032

8 adjustment disorder*.tw,kf. 2144

9 (affective disorder* or affective symptoms or anhedoni* or depressed or depression or depressive or dysphori* or dysthymi* or melanchol* or MDD or mood disorder* or low mood).tw,kf. 585974

10 (agoraphobi* or phobi* or anxiety disorder? or social* anxi* or body dysmorphi* or GAD or compulsi* or OCD or health anxiety or neurotic or neuros* or obsess* or panic or PND or ((sever* or serious* or major* or chronic* or complex* or critical* or endur* or persist* or resist* or acute) adj2 (anxiety or fear or worry or worries or mental)) or ADNOS).tw,kf. 320729

11 ((eat* adj3 disorder) or anorexi* or bulimi* or EDNOS or bing* eat* or (bing* and purg*)).tw,kf. 54892

12 (emotional trauma or ((post-trauma* or posttrauma* or post trauma*) adj stress*) or flashback* or (trauma* adj (avoidance or grief or nightmare* or stress)) or ((psych* or emotional) adj (stress or distress or trauma*)) or psychotrauma* or psycho-trauma* or PTSD or social stress or stress reactions or ((acute* or chronic* or extreme*) adj stress*) or DESNOS).tw,kf. 140698

13 (medical* unexplained or MUPs or somatoform or multisomatoform or somati#ation or hysteri* or somatic symptom? or functional disorder? or neurastheni* or conversion disorder* or hypochondria*).tw,kf. 32170

14 (auto mutilat* or automutilat* or (self adj (destruct* or harm* or immolat* or inflict* or injur* or mutilat* or poison*)) or selfdestruct* or selfharm* or selfimmolat* or selfinflict* or selfinjur* or selfmutilat* or selfpoison* or suicid* or parasuicid*).tw,kf. 113920

15 (personality disorder* or impulse control disorder*).tw,kf. 25465

16 (((emotional or mental or psychological or psychosocial or social) and (wellbeing or well-being or health)) or (emotion* adj2 (difficult* or disorder? or distress or stress* or regulat* or symptom* or outcome?))).tw,kf. 670970

17 ((bipolar adj3 (affective or depress* or disorder* or episode* or mood or psychosis or spectrum or state or states)) or cyclothymi* or affective psycho* or mania or manic or hypermani* or hypomani* or rapid cycling).tw,kf. 57843

18 (schizo* or psychotic or psychos* or paranoid or paranoia or delusions or delusional).tw,kf. 373981

19 (((drug? or substance?) adj3 (abuse* or addict* or dependen* or disorder* or misus* or users)) or (SUD or SUDs) or ((drug or substance) adj use*) or ((illicit or party or recreational or street) adj drug?) or ((inhalant or solvent*) adj3 (abuse* or addict* or dependen* or misus* or use*)) or (alcohol* adj3 (abuse* or addict* or dependen* or disorder* or misus*)) or (alcohol use* or alcoholism or alcholic* or alcohol* intoxication)).mp. or (binge drink* or (problem* adj2 (drink* or alcohol* use*))).tw,kf. 418376

20 or/1-19 3047865

21 exp Medical Students/ 44644

22 ((student? or graduate? or undergraduate? or postgraduate?) adj2 (medical or medicine or doctor)).tw,kf. 78533

23 exp Students, Health Occupations/ 89513

24 (Health* adj2 (student* or undergraduate? or graduate? or postgraduate?)).tw,kf. 18256

25 21 or 22 or 23 or 24 150369

26 exp Help-Seeking Behavior/ 1217

27 exp Disclosure/ 34879

28 exp Health Services Accessibility/ 136129

29 exp Self-Disclosure/ 7351

30 ((health or depression or anxiety or mental or psych*) adj1 literacy).tw,kf. 15799

31 disclosure.tw,kf. 25441

32 (health adj1 (behavior or behaviour)).tw,kf. 18525

33 (helpseek* or ((utili* or access* or use* or using or seek* or getting or barrier* or facilitat*) adj3 (service* or care or help or treat* or healthcare or health care or support))).tw,kf. 806708

34 26 or 27 or 28 or 29 or 30 or 31 or 32 or 33 988744

35 20 and 25 and 34 2492

36 limit 35 to yr="2013 -Current" 1895

**************************************

**Ovid APA PsycInfo** <1806 to January Week 2 2024>

1 health care seeking behavior/ or help seeking behavior/ or self-referral/ 13114

2 self-disclosure/ 8701

3 (self disclos* or selfdisclos* or self referr* or selfreferr* or (volunatry adj (disclos* or report* or referr*))).tw,id. 8458

4 (helpseek* or (help* adj3 seek*)).tw,id. 18652

5 Healthcare Utilization/ or Healthcare Access/ or Health Service Needs/ 7939

6 Treatment Barriers/ 7619

7 ((utili* or access* or use* or using or seek* or getting or barrier* or facilitat*) adj3 (service* or care or help or treat* or healthcare or health care or support)).tw,id. 225467

8 health literacy/ or mental health literacy/ 4717

9 ((health or depression or anxiety or mental or psych*) adj literacy).tw,id. 6133

10 or/1-9 252503

11 medical students/ 15260

12 ((student? or graduate? or undergraduate? or postgraduate?) adj2 (medical or medicine or doctor)).tw,id. 20430

13 11 or 12 23189

14 10 and 13 1298

15 (health* adj2 (student* or undergraduate? or graduate? or postgraduate?)).tw,id. 9424

16 (1 or 2 or 3 or 4) and 15 237

17 exp *college students/ or college graduates/ or graduate students/ or postgraduate students/ 84454

18 1 and 17 624

19 14 or 16 or 18 2081

20 limit 19 to yr="2013 -Current" 1230

**************************************

**Ovid Embase** <1974 to 2024 January 16>

1 exp mental disease/ 2752201

2 mental health/ 217281

3 *anxiety/ or social anxiety/ 66915

4 mental stress/ or physiological stress/ or chronic unpredictable stress/ 176058

5 (mental* or psychiatr* or psychopathol*).tw,kf. 976346

6 (adjustment disorder* or affective disorder* or affective symptoms or anhedoni* or depressed or depression or depressive or dysphori* or dysthymi* or melanchol* or MDD or mood disorder* or low mood).tw,kf. 794286

7 (agoraphobi* or phobi* or anxiety disorder? or social* anxi* or body dysmorphi* or GAD or compulsi* or OCD or health anxiety or neurotic or neuros* or obsess* or panic or PND or ((sever* or serious* or major* or chronic* or complex* or critical* or endur* or persist* or resist* or acute) adj2 (anxiety or fear or worry or worries or mental)) or ADNOS).tw,kf. 429763

8 exp eating disorder/ 66076

9 ((eat* adj3 disorder) or anorexi* or bulimi* or EDNOS or bing* eat* or (bing* and purg*)).tw,kf. 75973

10 (emotional trauma or ((post-trauma* or posttrauma* or post trauma*) adj stress*) or flashback* or (trauma* adj (avoidance or grief or nightmare* or stress)) or ((psych* or emotional) adj (stress or distress or trauma*)) or psychotrauma* or psycho trauma* or PTSD or social stress or stress reactions or ((acute* or chronic* extreme*) adj stress*) or DESNOS).tw,kf. 169336

11 functional disease/ 29018

12 (medical* unexplained or MUPs or somatoform or multisomatoform or somati#ation or hysteri* or somatic symptom? or functional disorder? or functional diease? or neurastheni* or conversion disorder* or hypochondria*).tw,kf. 42391

13 (auto mutilat* or automutilat* or (self adj (destruct* or harm* or immolat* or inflict* or injur* or mutilat* or poison*)) or selfdestruct* or selfharm* or selfimmolat* or selfinflict* or selfinjur* or selfmutilat* or selfpoison* or suicid* or parasuicid*).tw,kf. 144692

14 (personality disorder* or impulse control disorder*).tw,kf. 35338

15 (((emotional or mental or psychological or psychosocial or social) adj5 (wellbeing or well-being or health)) or (emotion* adj2 (difficult* or disorder? or distress or stress* or regulat* or symptom* or outcome?))).tw,kf. 514693

16 ((bipolar adj3 (affective or depress* or disorder* or episode* or mood or psychosis or spectrum or state or states)) or cyclothymi* or affective psycho* or mania or manic or hypermani* or hypomani* or rapid cycling).tw,kf. 84443

17 (schizo* or psychotic or psychos* or paranoid or paranoia or delusions or delusional).tw,kf. 486932

18 substance abuse/ 59151

19 (((drug? or substance?) adj3 (abuse* or addict* or dependen* or disorder* or misus* or users)) or (SUD or SUDs) or ((drug or substance) adj use*) or ((illicit or party or recreational or street) adj drug?) or ((inhalant or solvent*) adj3 (abuse* or addict* or dependen* or misus* or use*)) or (alcohol* adj3 (abuse* or addict* or dependen* or disorder* or misus*)) or (alcohol use* or alcoholism or alcholic* or alcohol* intoxication)).mp. or (binge drink* or (problem* adj2 (drink* or alcohol* use*))).tw,kf. 699102

20 or/1-19 4319748

21 health student/ or exp medical student/ or exp paramedical student/ or premedical student/ or public health student/ 145698

22 ((student? or graduate? or undergraduate? or postgraduate?) adj2 (medical or medicine or doctor)).tw,kf. 102012

23 (Health* adj2 (student* or undergraduate? or graduate? or postgraduate?)).tw,kf. 21715

24 or/21-23 202091

25 help seeking behavior/ 16268

26 voluntary reporting/ 1059

27 health care access/ or right to health/ or unmet medical need/ 93549

28 self disclosure/ 5078

29 ((health or depression or anxiety or mental or psych*) adj1 literacy).tw,kf. 20295

30 (helpseek* or ((utili* or access* or use* or using or seek* or getting or barrier* or facilitat*) adj3 (service* or care or help or treat* or healthcare or health care or support))).tw,kf. 1139914

31 or/25-30 1226022

32 20 and 24 and 31 2938

33 limit 32 to yr="2013 -Current" 2321

**********************************

**Web of Science Core Collection (all databases)**

#1 (“mental disorder*” or “mental health” or “mental* ill*” or “mental* well*” or psychiatr* or psychopathol* or psycho-pathol* or “adjustment disorder*” or “affective disorder*” or “affective symptoms” or anhedoni* or depressed or depression or depressive or dysphori* or dysthymi* or melanchol* or MDD or “mood disorder*” or “low mood” or agoraphobi* or phobi* or “anxiety disorder*” or “social* anxi*” or “body dysmorphi*” or GAD or compulsi* or OCD or “health anxiety” or neurotic or neuros* or obsess* or panic or PND or ((sever* or serious* or major* or chronic* or complex* or critical* or endur* or persist* or resist* or acute) near/2 (anxiety or fear or worry or worries or mental)) or ADNOS or (eat* near/3 disorder) or anorexi* or bulimi* or EDNOS or “bing* eat*” or (bing* and purg*) or “emotional* trauma*” or “post-trauma* stress” or “posttrauma* stress” or “post trauma* stress*” or flashback* or “trauma avoidance” or “trauma* grief” or (trauma* and nightmare*) or “trauma* stress” or “psych* stress*” or “psych* distress*” or “psych* trauma*” or “emotional* stress*” or “emotional* distress*” or “emotional* trauma*” or psychotrauma* or psycho-trauma* or PTSD or “social stress” or “stress reactions” or “acute* stress*” or “extrem* stress*” or DESNOS) (Topic) (1,789,419)

#2 (“medical* unexplained” or MUPs or somatoform or multisomatoform or somatisation or somatization or hysteri* or “somatic symptom*” or “functional disorder*” or neurastheni* or “conversion disorder*” or hypochondria* or “auto mutilat*” or automutilat* or “self destruct*” or “self harm*” or “self immolat*” or “self inflict*” or “self injur*” or “self mutilat*” or “self poison*” or selfdestruct* or selfharm* or selfimmolat* or selfinflict* or selfinjur* or selfmutilat* or selfpoison* or suicid* or parasuicid* or “personality disorder*” or “impulse control disorder*” or bipolar or cyclothymi* or “affective psycho*” or mania or manic or hypermani* or hypomani* or “rapid cycling” or schizo* or psychotic or psychos* or paranoid or paranoia or delusions or delusional) (Topic) (825,118)

#3 ((drug* near/3 (abuse* or addict* or dependen* or disorder* or misus* or users)) OR (substance* near/3 (abuse* or addict* or dependen* or disorder* or misus* or users)) OR SUD or SUDs or “drug use*” or “substance use*” or “illicit drug*” or “party drug*” or “recreational drug*” or “street drug*” or (inhalant* near/3 (abuse* or addict* or dependen* or misus* or use*)) or (solvent* near/3 (abuse* or addict* or dependen* or misus* or use*)) or (alcohol* near/3 (abuse* or addict* or dependen* or disorder* or misus*)) or “alcohol use*” or alcoholism or alcholic* or “alcohol* intoxication” or “binge drink*” or (problem* near/2 (drink* or alcohol*))) (Topic) (433,630)

#4 ((emotional or mental or psychological or psychosocial or social) near/5 (wellbeing or well-being or health)) or (emotion* near/2 (difficult* or disorder* or distress or stress* or regulat* or symptom* or outcome*)) (Topic) (582,161)

#5 (#1 OR #2 OR #3 OR #4) (2,748,796)

#6 ((“health* student*” or “health* graduate*” or “health* undergraduate*” or “health* postgraduate*” or “medic* student*” or “medic* graduate*” or “medic* undergraduate*” or “medic* postgraduate*” or “student medic*” or “graduate medic*” or “undergraduate medic*” or “postgraduate medic*” or “medic* school student*” or “medic* school graduate*” or “medic* school undergraduate*” or “medic* school postgraduate*” or “student doctor*” or “graduate doctor*” or “undergraduate doctor*” or “postgraduate doctor*”) SAME (helpseek* or (seek* and help*) or disclosure* or “self disclos*” or selfdisclos*)) (Topic) (964)

#7 ((student* or graduate* or undergraduate* or postgraduate*) AND (helpseek* or (seek* and help*) or disclosure* or “self disclos*” or selfdisclos*)) (Title) (870)

#8 ((“health* student*” or “health* graduate*” or “health* undergraduate*” or “health* postgraduate*” or “medic* student*” or “medic* graduate*” or “medic* undergraduate*” or “medic* postgraduate*” or “student medic*” or “graduate medic*” or “undergraduate medic*” or “postgraduate medic*” or “medic* school student*” or “medic* school graduate*” or “medic* school undergraduate*” or “medic* school postgraduate*” or “student doctor*” or “graduate doctor*” or “undergraduate doctor*” or “postgraduate doctor*”) NEAR/5 (utili* or access* or use* or using or seek* or getting or barrier* or facilitat*) NEAR/5 (service* or care or help or treated or treatment* or healthcare or “health care” or support)) (Topic) (681)

#9 ((“health* student*” or “health* graduate*” or “health* undergraduate*” or “health* postgraduate*” or “medic* student*” or “medic* graduate*” or “medic* undergraduate*” or “medic* postgraduate*” or “student medic*” or “graduate medic*” or “undergraduate medic*” or “postgraduate medic*” or “medic* school student*” or “medic* school graduate*” or “medic* school undergraduate*” or “medic* school postgraduate*” or “student doctor*” or “graduate doctor*” or “undergraduate doctor*” or “postgraduate doctor*”) SAME (“health literacy” or “depression literacy” or “anxiety literacy” or “mental* literacy” or “psych* literacy”)) (Topic) (229)

#10 (#6 OR #7 OR #8 OR #9) (2,529)

#11 (#5 AND #10) (966)

**************************************

**EBSCOhost Education databases**

*British Education Index (BEI), Education Resources Information Center (ERIC), Education Abstracts*

S1 TI ((student* or graduate* or undergraduate* or postgraduate*) N2 (medical or medicine or doctor)) OR AB ((student* or graduate* or undergraduate* or postgraduate*) N2 (medical or medicine or doctor)) (12,828)

S2 TI ((student* or graduate* or undergraduate* or postgraduate*) N2 health*)) OR AB ((student* or graduate* or undergraduate* or postgraduate*) N2 health*)) (12,246)

S3 (S1 OR S2) (24,464)

S4 (helpseek* or (help* N3 seek*)) (7,273)

S5 (S3 AND S4) (250)

S6 TI ("self disclos*" or selfdisclos* or "self referr*" or selfreferr* or "volunat* disclos*" or "volunt* report*" or "volunt* referr*") OR AB("self disclos*" or selfdisclos* or "self referr*" or selfreferr* or "volunat* disclos*" or "volunt* report*" or "volunt* referr*") (2.105)

S7 (S3 AND S6) (15)

S8 TI ( "health literacy" or "depression literacy" or "anxiety literacy" or "mental literacy" or "psych* literacy" ) OR AB ( "health literacy" or "depression literacy" or "anxiety literacy" or "mental literacy" or "psych* literacy") (1,462)

S9 (S3 AND S8) (182)

S10 (S5 OR S7 OR S9) (428)

S11 (S5 OR S7 OR S9) Limiters - Publication Date: 20130101-20240116 (341)

S12 TI ((utili* or access* or use* or using or seek* or getting or barrier* or facilitat*) N3 (counsel* or “mental health service*” or psychotherap* or psychiatri* or therapy or therapies or treatment* or healthcare or "health care")) OR AB ((utili* or access* or use* or using or seek* or getting or barrier* or facilitat*) N3 (counsel* or “mental health service*” or psychotherap* or psychiatri* or therapy or therapies or treatment* or healthcare or "health care")) Limiters - Publication Date: 20130101-20240116 (10,809)

S13 (S3 AND S12) Limiters - Publication Date: 20130101-20240116 (487)

**S14** (mental* or psychiatr* or psychopathol* or psycho-pathol* or psychological or psychosocial or wellbeing or well-being or “adjustment disorder*” or “affective disorder*” or “affective symptoms” or anhedoni* or depressed or depression or depressive or dysphori* or dysthymi* or melanchol* or MDD or mood or mood or agoraphobi* or phobi* or anxiety or “body dysmorphi*” or GAD or compulsi* or OCD or neurotic or neuros* or obsess* or panic or fear or worry or worries or ADNOS or “eating disorder*” or anorexi* or bulimi* or EDNOS or “bing eating” or binging or trauma* or post-trauma* or posttrauma* or “post trauma*” or flashback* or grief or nightmare* or stress or distress or emotion* or psychotrauma* or psycho-trauma* or PTSD or DESNOS or “medically unexplained” or MUPs or somatoform or multisomatoform or somatisation or somatization or hysteri* or “somatic symptom*” or “functional disorder*” or neurastheni* or “conversion disorder*” or hypochondria* or “auto mutilat*” or automutilat* or “self destruct*” or “self harm*” or “self immolat*” or “self inflict*” or “self injur*” or “self mutilat*” or “self poison*” or selfdestruct* or selfharm* or selfimmolat* or selfinflict* or selfinjur* or selfmutilat* or selfpoison* or suicid* or parasuicid* or “personality disorder*” or PND or “impulse control disorder*” or bipolar or cyclothymi* or “affective psycho*” or mania or manic or hypermani* or hypomani* or “rapid cycling” or schizo* or psychotic or psychos* or paranoid or paranoia or delusions or delusional or (drug* or substance* or SUD or SUDs or inhalant* or solvent* or alcohol* or “binge drink*” or “problem* drink*” or alcohol*) Limiters - Publication Date: 20130101-20240116 (260,149)

S15 (S13 AND S14) (318)

S16 (S11 AND S14) (255)

S17 (S15 OR S16) (398) [de-duplicated across all three databases]

*******************************************************************************

**Appendix 2: Interventions described according to the TIDieR Framework**

**Table 4 Intervention Design and Characteristics (according to TIDieR Framework)**

| **Psychiatry Clinical Clerkships** – Note that the control groups for the Newton-Howes study (described below) are included here as they are standard psychiatry clinical clerkships | | | | | | | | |
| --- | --- | --- | --- | --- | --- | --- | --- | --- |
|  | Almadani (2023) | | Zavorotnyy (2023) | | | | Newton-Howes (2021) Control Group | |
| Intervention name | 4 week Psychiatry Clinical Clerkship | | Psychiatry Clinical Clerkship | | | | 4-5 week clinical placement | |
| Why | Provide compulsory clinical experience in psychiatry | | Clinical experience and psychiatry education | | | | Control group (placement developed without co-producation) | |
| Materials | Psychiatry lectures. Placement in clinical setting | | Lectures, case discussions, journal clubs. Clinical clerkship (ward based with psychotherapy opportunities). | | | | Placement in clinical setting | |
| What and how | 2 weeks of large group face to face lectures on various psychiatry topics (focus on patient care rather than self-care). 2 weeks of clinical exposure through attendance at psychiatric clinics and clinical rounds | | 2 week clerkship with morning of clinical experience, focused on patient contact including following a patients’ care. Afternoon of taught sessions and case discussion on psychiatric topics. Student case presentation. | | | | 4-5 week apprenticeship style clinical placements. Focus on patient contact alongside a didactic teaching course. | |
| Who provided | Medical faculty and clinical staff* | | Clinical staff, medical faculty | | | | Clinical staff | |
| Where | University, Psychiatric Clinical settings | | Clinical setting, psychiatry department of medical school | | | | Psychiatric Clinical setting | |
| When and how often | 4 Weeks during medical degree | | 2 week psychiatry clinical clerkship | | | | 4-5 week clinical placement | |
| Variation | No modifications reported | | No modifications reported | | | | No modifications reported | |
| Fidelity | Not reported | | Mean 8.8 / 10 days attended | | | | Not reported | |
| **Interventions with a Lived Experience Element** | | | | | | | | |
|  | Fernandez (2016) | Jarvie (2013) | | Martin (2020) | | Newton-Howes (2021) Intervention Group | | Smith (2016) |
| Intervention name | One-off 3 hour Educational Intervention | One-off 2 ½ hour educational intervention with comedian | | One-off Lived Experience Educational Intervention | | “World of Difference” service-user led anti-stigma program delivered during psychiatry placement | | Anti-stigma campaign with educational sessions, and optional screening |
| Why | Educate about mental illness and reduce associated stigma | Reduce stigma around mental illness | | Reduce stigma around mental illness | | Reduce stigma around mental illness | | Reduce stigma, promote help seeking, encourage recognition mental illness |
| Materials | Lecture theatre. Personal testimony of experience of severe mental illness (video or face-to-face). | Lecture theatre, breakout rooms, comedians with lived experience of mental illness. | | Teleconference with breakout rooms. Physicians offering personal testimony of “vulnerability” (exam failure, immigration stress, mental illness) | | Workshop, clinical placement, tutorial, recommended reading list. | | Friends and Family session, email PowerPoint with audio recording, video of senior students with personal testimony of help seeking. |
| What and how | Lecture about mental illness, including discussion of stigma. Personal testimony around lived experience of mental illness delivered either live, or through screening of pre-recorded video | Lecture from founder of charity which trains people mental health problems in stand up comedy which included clips of ‘Cracking Up’ documentary. Small group interaction with people with stand-up comedy experience and a lived experience of mental illness. | | Panel of three physicians shared personal experience of vulnerability, then small group discussions which physicians joined. Curated materials recommended for further reading | | During psychiatry placement: Service user led interactive workshop (discussing stigma and recovery), Placement within service user led recovery service about which reflective assessment was completed. Optional tutorial to support reflective writing assessment. | | Anti-stigma campaign including: Friends and Family of Medical Students session, video normalizing and encouraging help-seeking which includes senior students with lived experience of seeking mental health support, online mental health screening tool |
| Who provided | Medical Faculty, Person with lived experience of serious mental illness who is in recovery | Founder of ‘Stand up for Mental Health’ charity, comedians with lived experience of mental illness. | | Medical Faculty | | Service users led workshop and hosting of students on placement. Educator delivered optional tutorial. | | Director of Student Development. Near peers with experience of helpseeking |
| Where | University | University campus, with lecture broadcast to other university site | | Online via teleconferencing software | | University, Psychiatric Clinical setting | | F&F session at F2F at university, and asynchronously via powerpoint with audio. Video. Online screening tool |
| When and how often | One-off session: 90 minute lecture, 40-45 minute personal testimony, over 3 hours | One off session: 90 minute lecture, 60 minute small group session | | One off session: 1 hour panel, then small groups (duration not reported). | | Over 4-5 week clinical placement: 6 hour workshop, 1 day clinical placement, optional 1 hour tutorial | | F&F session provided and video and screening tool launched at start of academic year (and available thereafter). |
| Variation | No modifications reported | No modifications reported | | Delivered online to students, some of whom were in different countries due to COVID restrictions. | | No modifications reported | | No modifications reported |
| Fidelity | 100% attendance | Not reported | | Not reported | | Reports that optional tutorials “poorly attended” | | Only 2 people completed online screening. |
| **Educational interventions without a lived experience element** | | | | | | | | |
|  | Takahashi (2022) | | | | Kurki (2023) | | | |
| Intervention name | 3 hour educational session including didactic teaching and role play | | | | Educational program to aid transition to university | | | |
| Why | Reduce stigma and improve MH literacy and support development of crisis management skills | | | | Facilitate transition to university through supporting mental health literacy and independent living. | | | |
| Materials | Lectures with worksheet (with blanks for completion). Gatekeeper training film, breakout groups of 3 people for role play exercises. | | | | Lectures. Online program translated and culturally adapted from Canadian transitions program **. Theory and Practice of Mindfulness audiotapes | | | |
| What and how | Lecture covering stigma, MH literacy, crisis management. Gatekeeper training film screening. Listening exercise. Lecture on crisis management. Role play of talking to student with self-stigma or suicidal thoughts. | | | | Two lectures introducing independent living, study and relationship skills, mental health literacy, and stress management. Online resources (with further detail including help seeking information). Mindfulness practice audiotapes made available. | | | |
| Who provided | Medical Faculty (psychiatrists and psychologist), Video by Ministry of Health, Labour and Welfare of Japan | | | | Medical faculty, mindfulness audiotapes by a mindfulness  instructor | | | |
| Where | University | | | | University, online, via audiotape | | | |
| When and how often | 1 hour lecture, 1 hour crisis management skills including gatekeeper film screening (13 minutes) and lecture, 1 hour listening exercise and role play. Delivered once. | | | | Two 60 minute lectures. No online time requirement. 20 mindfulness audiotape sessions 4-30 minute duration. | | | |
| Variation | No modifications reported | | | | No modifications reported | | | |
| Fidelity | Not reported | | | | Median online resource use 96.0 minutes, primarily spent on independent living skills, not MH literacy. | | | |
| **Interventions to increase awareness, uptake or availability of mental health support** | | | | | | | | |
|  | Williams (2018) | | | | Seritan (2014) | | | |
| Intervention name | Informative email offering an appointment with student health service | | | | Student wellbeing service providing psychological support, screening and non-targeted education. | | | |
| Why | Inform students about available MH service and encourage help seeking | | | | Improve service accessibility and availability, promote mental health literacy and self care, encourage recognition mental illness, change culture. | | | |
| Materials | Email, student mental health clinic appointment availability | | | | Mental health service (including psychologists, psychiatrist). Maslach Burnout Inventory (MBI). Workshops. Brochure for families. Online groups. | | | |
| What and how | Email sent detailing available MH services and offering appointment with student MH service. Students requesting appointments contacted to arrange these within 24 hours, and seen within 1 week | | | | Expansion of healthcare service to offer:   - Psychiatrist input - Urgent care - Triage for students with MH difficulty   Screening with MBI  Workshops for students, their families, and faculty members covering breadth of self-care, wellbeing and career relevant topics.  Peer support and student groups | | | |
| Who provided | Student mental health service staff | | | | Psychologists and psychiatrists provide clinical care. Associate dean of student wellness oversees intervention | | | |
| Where | Email | | | | University, online, brochures, clinical centre on campus | | | |
| When and how often | Single email offering information and appointment with student mental health. | | | | Clinical centre available all year. Regular workshops. Annual screening with MBI. | | | |
| Variation | No modifications reported | | | | No modifications reported | | | |
| Fidelity | Not reported | | | | Example workshop for friends and family had 30 – 78% attendance | | | |

*Clinical staff refers to staff whose primary aim is the care of the patients who students may see in a professional capacity (as opposed to educating or treating students).

**Appendix 3: Risk of Bias Assessments**

**Table 5: Risk of Bias assessment for individual studies**

| **ROB2 Risk of Bias Assessment** | | | | | | | | | | | | | | | | |
| --- | --- | --- | --- | --- | --- | --- | --- | --- | --- | --- | --- | --- | --- | --- | --- | --- |
|  | |  | | ROB2 Domain: Risk of bias due to... | | | | | | | | | | | | |
| Author | | Study Type | | D1 the randomization process | | D2 deviations from the intended interventions | | D3 missing outcome data | | | D4 measurement of the outcome | | D5 selection of the reported result | | Overall risk of bias Judgement | |
| Fernandez (2016) | | RCT with two active arms. Compulsory course. 91.9% participated in study. | | Low | | Low | | Low | | | Low | | Some Concerns | | Some Concerns | |
|  | |  | | No registered protocol available therefore analysis plan may not have been finalized prior to unblinded outcome data becoming available to the trial investigators. However no evidence of multiple analyses or measures of help-seeking. | | | | | | | | | | | | |
| **ROBINS I (V2) adapted Risk of Bias Assessment** | | | | | | | | | | | | | | | | |
|  |  | | ROBINS I (V2) Domain: Risk of bias due to... | | | | | | | | | | | | | |
| Author | Study Type | | D1 Confounding | | D2 Classification of Intervention | | D3 Selection of Participants | | D4 Deviations form intended intervention | D5 Missing Data | | D6 Measurement of outcome | | D7 Selection of reported result | | Overall risk of bias Judgement |
| Almadani (2023) | Cross sectional survey comparing students who had / had not done psychiatry placement. All students do placement and 95.6% students surveyed responded | | Serious | | Low | | Low | | Low | Low | | Moderate | | Low | | Serious |
|  |  |  | Students in different years of course compared. Therefore student experience (outside of psychiatry) will have been different and seriously confound relationship. Evaluation occurred at variable time after intervention occurred. Knowledge of the assigned intervention could have influenced participant-reported outcomes but probably only to a small extent. Results presented for individual items on OMS-HC scale and domain level scores (thus multiple measures of help-seeking) but no suggestion results were being presented selectively. | | | | | | | | | | | | | |
| Fernandez (2016)* for pre-post data (see RoB 2 for evaluation bias in RCT) | Pre-post data extracted from an RCT with two active arms. (RoB2 evaluation for the RCT itself is presented above). Compulsory course. 91.9% participated in study. | | Moderate | | Low | | Low | | Low | Low | | Moderate | | Low | | Moderate |
|  |  |  | Considering pre-post evaluation (not RCT): Possible confounding effect of time (though very brief interval so unlikely to be significant). Knowledge of the assigned intervention could have influence participant-reported outcomes but probably only to a small extent. | | | | | | | | | | | | | |
| Jarvie (2013) | Before-after study. 37.7% of course attendees consented to participation in the research, all completes follow up measures | | Moderate | | Low | | Low | | Low | Low | | Moderate | | Low | | Moderate |
|  |  |  | Possible confounding effect of time (though very brief interval so unlikely to be significant). Knowledge of the assigned intervention could have influence participant-reported outcomes but probably only to a small extent. | | | | | | | | | | | | | |
| Kurki (2023) | Before-after study. Optional course (58.8% those eligible undertook course), of whom 71.8% completed follow up measures. | | Serious | | low | | low | | Low | serious | | Moderate | | low | | serious |
|  |  |  | Confounding effect of time during transition to university. Missing data with no information as to whether non-completion of follow up measures was due to lack of intervention effect. Knowledge of the assigned intervention could have influenced participant-reported outcomes but probably only to a small extent. | | | | | | | | | | | | | |
| Martin (2020) | Before-after study. Compulsory course. 87% of attendees completed follow up measures ** | | Serious | | Low | | Low | | Low | Serious** | | Moderate | | Low | | Serious |
|  |  |  | Confounding effect of time with unclear duration between baseline and follow-up measures, and uncertainty whether other taught components of the course occurred within this timeframe. Missing data, with no information as to whether non-completion of follow up measures was due to lack of intervention effect. Knowledge of the assigned intervention could have influenced participant-reported outcomes but probably only to a small extent. | | | | | | | | | | | | | |
| Newton-Howes (2021) | Controlled study. Compulsory course. Approximately half of students completed follow up measures ** | | Serious | | Low | | Low | | Low | Serious** | | Moderate | | Low | | Serious |
|  |  |  | Control group received placement at different site introducing confounding (different environment, clinical supervisors). Missing data with no information as to whether non-completion of follow up measures was due to lack of intervention effect. Knowledge of the assigned intervention could have influenced participant-reported outcomes but probably only to a small extent. | | | | | | | | | | | | | |
| Seritan (2014) | Interrupted time series. | | Serious | | Low | | Low | | Low | Low | | Low | | Low | | Serious |
|  |  |  | Confounding effect of time (including societal changes in stigma around help seeking for mental illness). A lack of evaluation of help-seeking from external resources at each time point means apparent increase in help seeking with intervention may well reflect change in provider (external to university provider) rather than change in actual help-seeking. | | | | | | | | | | | | | |
| Smith (2016) | Repeat cross-sectional study, before-after intervention. Data for 15 participants presented, of 68 at baselines. | | Serious | | low | | low | | Serious | Serious | | Moderate | | Low | | Serious |
|  |  |  | Confounding effect of time during transition to university for some participants. Missing data, with those who did not engage with intervention being deliberately excluded (not complete case analysis), and no information as to whether non-completion of follow up measures by others was due to lack of intervention effect. Knowledge of the assigned intervention could have influenced participant-reported outcomes but probably only to a small extent. Multiple measures of help-seeking but no suggestion results were being presented selectively. | | | | | | | | | | | | | |
| Takahashi (2022) | Before-after study. Compulsory course. 89% completed first follow up measure.** | | Moderate | | low | | low | | low | serious** | | Moderate | | low | | serious |
|  |  |  | Possible confounding effect of time (though very brief interval to first follow-up so unlikely to be significant). Missing data with no information as to whether non-completion of follow up measures was due to lack of intervention effect. Knowledge of the assigned intervention could have influenced participant-reported outcomes but probably only to a small extent. | | | | | | | | | | | | | |
| Williams (2018) | Healthcare utilization rates, pre-post intervention | | Serious | | low | | low | | low | low | | low | | Serious | | Serious |
|  |  |  | Confounding effect of time, with a lack of evaluation of help-seeking from external resources at each time point, meaning apparent increase in help seeking with intervention may reflect change in provider (external to university provider) rather than change in actual help-seeking. Multiple analyses of the data pertaining to service uptake presented in the results, introducing possible bias in the selection of reported results. | | | | | | | | | | | | | |
| Zavorotnyy (2023) | Before-after study. | | Moderate | | Low | | Low | | Low | Serious | | Moderate | | Low | | Serious |
|  |  |  | Possible confounding effect of time (though short interval without evidence of additional cofounding so unlikely to be significant). Missing data with evidence of similarity between completers and non-completers at baseline, but no information as to whether non-completion of follow up measures was due to lack of intervention effect. Knowledge of the assigned intervention could have influenced participant-reported outcomes but probably only to a small extent. | | | | | | | | | | | | | |

* This study was designed and conducted as an RCT with two active control groups and ROB2 risk of bias is provided above. Given both groups were active, we have also displayed the results for each arm (pre-post data). This ROBINSi score evaluates the risk of bias when evaluating each arm as a separate pre-post study (as is comparable to the majority of studies presented here), rather than evaluating the RCT.

** Unclear whether missing data due to eligible students declining to participate or drop out following commencement of the study therefore this may be an overestimate of risk of bias.

**Figure 5: Forest plot of the effect of lived experience interventions on medical students’ attitudes to help-seeking for personal mental health problems, based on data from before-after studies with a moderate risk of bias**

**Appendix 4: Sensitivity Analysis**

**Table 6: Effect Size ordered by Prevalence of MH problems**

| Study | Mental health problem or moderate (or greater) symptoms | Baseline Help-seeking Measure | Pre-post intervention SMD (SD) | Study Group |
| --- | --- | --- | --- | --- |
| Takahashi (2022) | Depression screen 1.1%^c, d^ | General Help-Seeking Questionnaire (GHSQ) 10.73 (4.62) | 0.571 (0.913) | 3 |
| Zavorotnyy (2023) | MH problem 6%^a^ | OMS-15 13.9 (3.7) | 0.501 (2.059) | 1 |
| Almadani (2023) | MH problem 13.6%^a^ | OMS-15 12.10 (3.12) | -0.052 (0.997) | 1 |
| Martin (2020) | MH problem 18%^a^  Depression screen 88.2% ^c, d^ | OMS-15 14.4 (3.1) | 0.462 (1.014) | 2 |
| Jarvie (2013) | MH problem 31% | OMS-20 3.05 (0.56) | 0.382 (1.231) | 2 |
| Kurki (2023) | MH problem 31%^a^  MH symptom screen 47.9% ^b, d^ | Other measure 21.6 (2.5 ^a^) | 0.198 (0.749) | 3 |

a SD calculated from SE and n

**Table 7: Effect Size ordered by Gender**

| **Study** | **Gender (% Male)** | **Baseline Help seeking measure** | **SMD (SD)** | **Study Group** |
| --- | --- | --- | --- | --- |
| Fernandez (2016) | 22% | OMS-15 HC 12.58 (0.37) | 0.873 (0.832) | 2 |
| Jarvie (2013) | 45% | OMS-20 HC 3.05 (0.56) | 0.382 (1.231) | 2 |
| Seritan (2014) | 46% | Proportion of students using service 32.1% (6.9) | 1.176 | 4 |
| Almadani (2023) | 51% | OMS-15 HC 12.10 (3.12) | -0.052 (0.997) | 1 |
| Martin (2020) | 54% | OMS-15 HC 14.4 (3.1) | 0.462 (1.014) | 2 |
| Takahashi (2022) | 65% | General Help-Seeking Questionnaire (GHSQ) 10.73 (4.62) | 0.571 (0.913) | 3 |
| Zavorotnyy (2023) | 65% | OMS-15 HC 13.9 (3.7) | 0.501 (2.059) | 1 |
| Kurki (2023) | 74% | Other measure 21.6 (2.5 ^a^) | 0.198 (0.749) | 3 |
