# Supplementary material for: Effect of medical school initiatives on help seeking for mental health problems among medical students: a systematic review and meta-analysis
Source: BMJ Open. 2026 Feb 9;16(2):e111351. doi: 10.1136/bmjopen-2025-111351 (PMC12887498; doi:10.1136/bmjopen-2025-111351)
Supplement: Supplementary file 4 [file bmjopen-2025-111351.reviewer_comments.pdf]

PEER REVIEW HISTORY

BMJ Open publishes all reviews undertaken for accepted manuscripts. Reviewers are asked to complete a checklist review form and are provided with free text boxes to elaborate on their assessment. These free text comments are reproduced below.

ARTICLE DETAILS

Title (Provisional)

The Effect of Medical School Initiatives on Help-Seeking for Mental Health Problems Among Medical Students: A Systematic Review and Meta-Analysis

Authors

Manley, Amy E; Perry, Rachel; Moran, Paul; Dawson, Sarah; Biddle, Lucy; Savović, Jelena

---

VERSION 1 - REVIEW

**Reviewer**	**1**
**Name**	**Sahu, Pradeep**
**Affiliation**	**University of the West Indies, Centre for Medical Sciences Education**
**Date**	**26-Oct-2025**
**COI**	**None**

This review addresses an important topic on institutional strategies to improve help-seeking among medical students. Kindly see my comments below for further improvement:

Abstract:

• The abstract would benefit from briefly mentioning the databases searched and inclusion/exclusion criteria to enhance transparency and reproducibility.

• The conclusion should acknowledge that the statistically significant improvements may have limited practical relevance due to small sample sizes and short follow-up periods.

Introduction:

• The introduction is clear and well structured, providing a strong rationale for the study and effectively highlighting the existing research gap and objectives.

Methodology:

• The methodology is well organized and largely compliant with best practices for systematic reviews.

Results:

• The results are clearly presented, with findings appropriately grouped by study design, intervention type, and outcome, which aids readability and comprehension.

Discussion:

• The discussion effectively summarizes the main findings; however, it could benefit from a clearer linkage between the results and existing literature, particularly in contextualizing why lived experience interventions may work better.

• The Discussion section should more explicitly analyze how the methodological limitations (e.g., low certainty, high risk of bias, small sample sizes) affect the interpretation of results.

• A concise conclusion summarizing the key findings, implications, and overall certainty of evidence should be added at the end of the Discussion.

References:

Follow the journal guideline for reference. For examples:

Dyrbye LN, Thomas MR, Power D V, et al (2010) Burnout and Serious Thoughts of

20 Dropping Out of Medical School: A Multi-Institutional Study. (You have written et al after three authors. Use the guideline.)

Dyrbye LN, Thomas MR, Shanafelt TD (2006) Systematic Review of Depression,

22 Anxiety, and Other Indicators of Psychological Distress Among U.S. and Canadian 23 Medical Students. Academic Medicine 81: (Incomplete reference)

**Reviewer**	**2**
**Name**	**McIntosh, Alasdair**
**Affiliation**	**University of Glasgow, Robertson Centre for Biostatistics**
**Date**	**29-Oct-2025**
**COI**	**None**

Thank you for your paper.

The paper would benefit from having the research objective clearly defined in the main paper. The objectives are mentioned in the abstract but they could also be made clear there too. Maybe these could be rephrased as a set of more concrete research questions. Do medical school initiatives significantly increase the rate of help seeking? If so, which ones are effective? Is the effect sustained over time? For each type of initiative, how large is the effect?

As the authors rightly acknowledge the evidence base in the area is weak and of low certainty with all but two of the studies having serious risk of bias. It is to authors credit that they have gone through the systematic review process in a principled way and identified

these problems. The high heterogeneity observed is unsurprising given the different approaches and outcome measures the studies covered use. As such, the meta analysis is of limited value to draw conclusions from. However, the manuscript does provide a potentially useful overview of the recent previous work in the area of interventions aimed at encouraging medical students to seek help for their mental health issues. The paper could be revised to shift the emphasis more onto this overview rather than the meta analysis.

Minor points-

Page 10 - new line needed for the "Synthesis Methods" title

Page 11 - Replace the "?" in "... therefore presented in tables ?" with the appropriate numbers.

Table 2 - p-values quoted here appear to have been copied from each individual paper. Please indicate in the footnotes which statistical test was used in each case.

Page 31 - "These limitations highlight the need rigorous..." Add "for" between "need" and "rigorous".

Reviewer 3

Name Cao, Wenhao

Affiliation University of Minnesota, Division of Biostatistics and Health Data Science

Date 02-Nov-2025

COI None

This is a well-conducted systematic review and meta-analysis that adheres to PRISMA and Cochrane guidelines. The authors have employed rigorous methods for study selection, data extraction, and risk of bias assessment. However, the evidence base is limited by the quality and heterogeneity of the included studies, which constrains the strength of the conclusions.

1. Most meta-analyses included only 2–5 studies, limiting the reliability of pooled estimates and the ability to assess publication bias via funnel plots. For some analysis, the I^2 is small and the author should consider using fixed-effect/common-effect) model rather than random effects model. Please check the guidance of this paper for meta-analysis with small number of studies (2-5). Bender, R., Friede, T., Koch, A., Kuss, O., Schlattmann, P., Schwarzer, G. and Skipka, G., 2018. Methods for evidence synthesis in the case of very few studies. Research synthesis methods, 9(3), pp.382-392.

2. Most studies were uncontrolled pre-post designs with serious risk of bias. The authors appropriately downgraded the certainty of evidence using GRADE.

Suggestion: Consider a sensitivity analysis excluding studies with the highest risk of bias, even if only a few remain.

3. The authors used the ROB-ME tool and concluded a low risk of publication bias for most analyses. Given the small number of studies, this conclusion should be interpreted cautiously.

Typo: 1. Page 12, line 11, "?" incorrectly used.

VERSION 1 - AUTHOR RESPONSE

Reviewer: 1
Dr. Pradeep Sahu, University of the West Indies

Comments to the Author:
This review addresses an important topic on institutional strategies to improve help-seeking among medical students. Kindly see my comments below for further improvement:

Abstract:
The abstract would benefit from briefly mentioning the databases searched and inclusion/exclusion criteria to enhance transparency and reproducibility.

These details have been added to the Abstract, as suggested **(page 2, lines 12-13):**

"Data Sources: MEDLINE Ovid, EMBASE Ovid, PsycINFO Ovid, Web of Science, ERIC, BEI, and Education Abstracts."

• **The conclusion should acknowledge that the statistically significant improvements may have limited practical relevance due to small sample sizes and short follow-up periods.**

Thank you, the following details have been added to the Discussion:

*"The small sample sizes and small number of studies included in the meta-analyses resulted in wide confidence intervals, leading to uncertainty about the direction of some effects and limiting conclusions about the size of any effect." **(page 33, lines 3-5)***

*"…the results from three studies which assessed longer-term impact on attitudes indicated that change is not sustained over time. These limitations constrain the practical relevance of the findings and highlight the need for rigorous evaluations to identify which initiatives are most effective and what frequency can foster lasting impact." **(page 33, lines 14-18)***

Introduction:

• The introduction is clear and well structured, providing a strong rationale for the study and effectively highlighting the existing research gap and objectives.

Methodology:

• The methodology is well organized and largely compliant with best practices for systematic reviews.

Results:

• The results are clearly presented, with findings appropriately grouped by study design, intervention type, and outcome, which aids readability and comprehension.

Thank you for this positive feedback about the introduction, methodology and results sections of this paper.

Discussion:

• The discussion effectively summarizes the main findings; however, it could benefit from a clearer linkage between the results and existing literature, particularly in contextualizing why lived experience interventions may work better.

We have expanded our discussion about why interventions may (or may not) be effective in modifying help seeking attitudes in this population, linking our findings with the existing literature, for example:

"Our very low certainty finding that standard psychiatry clerkships have little to no effect on help-seeking attitudes, contrasts with empirical and meta-analytical evidence, including from studies within this review, that clinical placements reduce medical students' stigma towards people with mental health problems [35,36,40,53,54]." **(page 34 line 21 to page 35 line 2).**

"contrary to findings in other populations [55], addressing stigma in medical students, may be insufficient to change help-seeking behaviour in this population" **(page 35 lines 3-4)**

"This finding resonates with literature showing that contact with people with mental health problems reduces stigma among healthcare professionals, thus promoting help seeking [53,56]." **(page 35 lines 12 to 14)**

• The Discussion section should more explicitly analyze how the methodological limitations (e.g., low certainty, high risk of bias, small sample sizes) affect the interpretation of results.

We expanded the "Strengths and Limitations" sections of the discussion to better highlight the limitations of our findings (page 32 line 15 to page 35 line 13) and used Cochrane specified language to present results to ensure the low to very low certainty of the evidence is clear.

• A concise conclusion summarizing the key findings, implications, and overall certainty of evidence should be added at the end of the Discussion.

This has been added **(page 36 line 14 to page 37 line 2).**

References:
Follow the journal guideline for reference. For examples:
Dyrbye LN, Thomas MR, Power D V, et al (2010) Burnout and Serious Thoughts of 20 Dropping Out of Medical School: A Multi-Institutional Study. (You have written et al after three authors. Use the guideline.)

Dyrbye LN, Thomas MR, Shanafelt TD (2006) Systematic Review of Depression, 22 Anxiety, and Other Indicators of Psychological Distress Among U.S. and Canadian 23 Medical Students. Academic Medicine 81: (Incomplete reference)

Thank you for identifying these issues. We recognise the importance of accurate referencing and have added the full citations (**see references pages 39 to 44).**

<u>Reviewer: 2</u>
Dr. Alasdair McIntosh, University of Glasgow
Comments to the Author:
Thank you for your paper.

The paper would benefit from having the research objective clearly defined in the main paper. The objectives are mentioned in the abstract but they could also be made clear there too. Maybe these could be rephrased as a set of more concrete research questions. Do medical school initiatives significantly increase the rate of help seeking? If so, which ones are effective? Is the effect sustained over time? For each type of initiative, how large is the effect?

We have followed your advice and have stated the objectives clearly at the end of the introduction **(page 6, lines 11-14).**

" we set out to establish whether initiatives delivered to unselected medical students, in a university setting, influence the rate of help-seeking for mental health problems among medical students. We sought to evaluate the direction of the effect and whether it was sustained over time."

As the authors rightly acknowledge the evidence base in the area is weak and of low certainty with all but two of the studies having serious risk of bias. It is to authors credit

that they have gone through the systematic review process in a principled way and identified these problems. The high heterogeneity observed is unsurprising given the different approaches and outcome measures the studies covered use. As such, the meta analysis is of limited value to draw conclusions from. However, the manuscript does provide a potentially useful overview of the recent previous work in the area of interventions aimed at encouraging medical students to seek help for their mental health issues. The paper could be revised to shift the emphasis more onto this overview rather than the meta analysis.

Thank you for your constructive feedback. We appreciate your acknowledgment of our rigorous systematic review process and our transparency in identifying the limitations of the existing evidence base. We understand the caution regarding the utility of the meta-analysis due to these weaknesses and that the low to very low certainty evidence necessitates a careful interpretation of the meta-analytic findings. To this end, we have expanded the discussion about the limitations (page 32 line 15 to page 35 line 13) and adhered to Cochrane mandated phrasing when discussing our findings, in order to highlight these limitations e.g. *"Educational initiatives and initiatives aiming to improve access to services may improve attitudes but the evidence is very uncertain"* **(conclusions page 36, lines 21-22).**

We believe the meta-analysis offers a robust approach to the quantification of the potential effect sizes for different intervention types, which, despite the low certainty, provides a benchmark for future research. We recognise that there is heterogeneity between interventions and have therefore ensured that studies are grouped according to intervention and study type, and used random effects models, for the meta-analyses. We also agree that a further narrative discussion may add additional value. To this end we have expanded our discussion of the findings in the context of the broader literature (**page 34, line 16 to page 36 line 13).** For those readers who may be looking to develop new interventions to increase help-seeking behaviour, we have provided additional detail about each of the interventions synthesised in the supplementary information, according to the TIDieR Framework **(supplementary material, appendix 2, pages 11 to 14 .**

Minor points-
Page 10 - new line needed for the "Synthesis Methods" title
Page 11 - Replace the "?" in "... therefore presented in tables ?" with the appropriate numbers.
Table 2 - p-values quoted here appear to have been copied from each individual paper. Please indicate in the footnotes which statistical test was used in each case.
Page 31 - "These limitations highlight the need rigorous..." Add "for" between "need" and "rigorous".

Thank you, these points have all been amended in the revised manuscript.

__Reviewer: 3__

Dr. Wenhao Cao, University of Minnesota

Comments to the Author:

This is a well-conducted systematic review and meta-analysis that adheres to PRISMA and Cochrane guidelines. The authors have employed rigorous methods for study selection, data extraction, and risk of bias assessment. However, the evidence base is limited by the quality and heterogeneity of the included studies, which constrains the strength of the conclusions.

1. Most meta-analyses included only 2–5 studies, limiting the reliability of pooled estimates and the ability to assess publication bias via funnel plots. For some analysis, the I^2 is small and the author should consider using fixed-effect/common-effect) model rather than random effects model. Please check the guidance of this paper for meta-analysis with small number of studies (2-5). Bender, R., Friede, T., Koch, A., Kuss, O., Schlattmann, P., Schwarzer, G. and Skipka, G., 2018. Methods for evidence synthesis in the case of very few studies. Research synthesis methods, 9(3), pp.382-392.

Thank you for this suggestion, and highlighting this paper. We pre-planned to use random effects meta-analysis for this study as our inclusion criteria were fairly broad, especially with regards to the intervention types and study designs. Given this anticipated heterogeneity in the interventions, we considered a random effects approach to be more appropriate, rather than a fixed effect method which assumes there is one true treatment effect across all included interventions, which we felt was implausible. This variety in interventions with resultant heterogeneity of findings (and larger I^2) in all meta-analyses except that shown in figure 2 would render fixed effects meta-analysis inappropriate. As this was pre-planned we have not changed to a fixed-effects approach as this would have been a post-hoc decision. However, on your advice we have now conducted a fixed-effect/common-effect for the meta-analysis of non-randomised controlled studies where the studies were sufficiently similar and homogenous to enable this. We have included this in the supplemental information **(see supplemental information, figure 5).** This did not change the findings. We also expanded out discussion of the limitations of fixed effects models with few studies.

"We prospectively opted to use random effects meta-analysis, given variation in help-seeking initiatives, which would make a single common treatment effect unlikely [51]. Nonetheless we recognise such models have been criticised for the difficulty in accurately estimating the

between-study variance when the number of included studies is small, which can affect the precision of the confidence intervals [51].”

(page 34, lines 8 to 13)

2. Most studies were uncontrolled pre-post designs with serious risk of bias. The authors appropriately downgraded the certainty of evidence using GRADE.

Suggestion: Consider a sensitivity analysis excluding studies with the highest risk of bias, even if only a few remain.

Thank you. We have included a sensitivity analysis in the supplementary material (figure 6) and referred to the analysis in the body of the article **(page 26, lines 7 - 11):**

“All but two [35] studies were deemed at serious risk of bias therefore sensitivity analysis excluding studies at serious risk of bias was only possible for lived experience initiatives. This did not substantially alter the findings, revealing a similar effect size for studies at moderate risk of bias (SMD 0.59; 95% CI 0.05 - 1.13; p=0.03; see supplementary material figure 6) as the overall analysis (see figure 3).“

It did not substantially change the SMD (though, given the limited number of studies the precision of the estimate was reduced).

3. The authors used the ROB-ME tool and concluded a low risk of publication bias for most analyses. Given the small number of studies, this conclusion should be interpreted cautiously.

Thank you for highlighting this. We have discussed our decisions using the RoB-ME tool for the outcome assessed in the review. The outcomes we were interested in for this review (help-seeking) were not the main focus of interest in the included studies. This led to a RoB-ME judgement of low risk of bias because, even if a study were unpublished because of the nature of the findings of its primary outcome, this would not necessarily equate to reporting bias for the help-seeking outcome which was of interest to us. However, we appreciate this does not mean that there aren’t any missing studies/results – it only means that we judged that any missing results are unlikely to be missing because of the nature of the result. We have thus added a statement that acknowledges potential for missing evidence:

"Nonetheless, given the limited number of studies and their observational nature, without registered protocols, there may be further similar unpublished studies." ***(Page 28, lines 7-9)***

Typo: 1. Page 12, line 11, "?" incorrectly used.

This has been corrected.

VERSION 2 - REVIEW

Reviewer 3

Name Cao, Wenhao

Affiliation University of Minnesota, Division of Biostatistics and Health Data Science

Date 20-Dec-2025

COI

All comments are addressed, No additional comments added
